# Genome-Wide Meta-Analysis Identifies Multiple Novel Rare Variants to Predict Common Human Infectious Diseases Risk

**DOI:** 10.3390/ijms24087006

**Published:** 2023-04-10

**Authors:** Andrea Gelemanović, Tatjana Ćatipović Ardalić, Ajka Pribisalić, Caroline Hayward, Ivana Kolčić, Ozren Polašek

**Affiliations:** 1 Department of Public Health, University of Split School of Medicine, 21000 Split, Croatia; 2 Department of Pediatrics, University Hospital of Split, 21000 Split, Croatia; 3 Medical Research Council Human Genetics Unit, Institute of Genetics and Cancer, University of Edinburgh, Western General Hospital, Edinburgh EH4 2XU, UK; 4 Department of General Courses, Algebra University College, 10000 Zagreb, Croatia

**Keywords:** genome-wide association study, rare variant, infection, hepatitis, meningitis, pneumonia, tuberculosis

## Abstract

Infectious diseases still threaten global human health, and host genetic factors have been indicated as determining risk factors for observed variations in disease susceptibility, severity, and outcome. We performed a genome-wide meta-analysis on 4624 subjects from the 10,001 Dalmatians cohort, with 14 infection-related traits. Despite a rather small number of cases in some instances, we detected 29 infection-related genetic associations, mostly belonging to rare variants. Notably, the list included the genes CD28, INPP5D, ITPKB, MACROD2, and RSF1, all of which have known roles in the immune response. Expanding our knowledge on rare variants could contribute to the development of genetic panels that could assist in predicting an individual’s life-long susceptibility to major infectious diseases. In addition, longitudinal biobanks are an interesting source of information for identifying the host genetic variants involved in infectious disease susceptibility and severity. Since infectious diseases continue to act as a selective pressure on our genomes, there is a constant need for a large consortium of biobanks with access to genetic and environmental data to further elucidate the complex mechanisms behind host–pathogen interactions and infectious disease susceptibility.

## 1. Introduction

Despite the extensive research on infectious diseases, we still face major limitations in understanding their pathogenesis. Uncertainties arise from a pathogen’s adaptability to modify or adapt to a new environment, different mechanisms of host–pathogen interactions, host genetics and possibly even random effects arising from interactions between a host and pathogen [1,2,3]. Recent studies often focus on the host’s genetic profile, which is suggested to be a determining factor in variations in disease occurrence and treatment outcome [4].

Previous studies often used candidate genes that are involved in the innate or adaptive immune response and play a role in a spectrum of infectious disease-associated outcomes [5]. Such studies achieved numerous positive results, with IL4, TLR2 and CCL5 validated in a large-scale systematic review and meta-analysis of respiratory infectious diseases [6]. The same study suggested numerous methodological barriers present in primary studies, suggesting that improvements are needed before translatable knowledge can be developed [6].

One frequently used study design in this situation is a hypothesis-free genome-wide association study (GWAS). The advantage of this design includes the possibility of discovering genes that were not previously implicated in disease pathogenesis, and thus understanding a much broader set of predictors for infectious diseases [7,8,9]. This study design can be especially effective in isolated populations, where allele frequencies might have drifted [10,11], as well as in the meta-analytic design [12,13], where the analysis of rare variants can also help to provide a better understanding of disease mechanisms [14]. Although the most common GWAS approach is still a univariate model where a single phenotype is associated with genetic variants, multivariate GWAS approaches that rely on associating several highly correlated phenotypes with genetic variants are recently gaining attention and could be used in addition to univariate models to increase their power [15,16,17]. In addition, to better understand better a particular genetic variant carries the risk for disease prediction, instead of the traditional GWAS approach, which focuses on each single genetic variant, there is an increased trend toward the use of various machine learning approaches, which is well suited for high-dimensional data and allows for the detection of the epistatic or non-linear effects among genetic variants with a particular phenotype [18]. However, there are only a few univariate GWA studies available for bacterial and viral infectious diseases, most of which are for tuberculosis, with modest replications between them [6,19,20,21,22,23,24,25,26,27]. The emergence of COVID-19 prompted even more research interest [28,29], promising possible new diagnostic and treatment options based on these discoveries [30,31,32,33]. Therefore, the aim of this study was to apply genome-wide meta-analysis to gain insight into bacterial and viral infectious disease pathogenesis and the possible role of host genetics in disease development.

## 2. Results

A total of 4624 participants were included in this study. No specific inclusion or exclusion criteria were established, except for age > 18 years. A substantial number of differences between the sub-cohorts were observed, both in the infection-related traits and in confounding variables, e.g., there were more women in the Korčula sub-cohort, slightly younger participants in the Split sub-cohort and participants in the Vis sub-cohort had in general a lower socioeconomic status and fewer years of schooling (Table 1). Due to the observed differences, demographic characteristics were used as confounding variables in the GWAS models.

The genome-wide meta-analysis of all three sub-cohorts showed 28 Bonferroni corrected genome-wide significant associations (*p* < 3.57 × 10^−9^) with hepatitis (six loci), meningitis (eight loci), systemic infections (seven loci) and tuberculosis (seven loci), while one locus showed suggestive association (*p* < 5 × 10^−8^) with pneumonia (Table 2). Manhattan and QQ plots for these traits are shown in Figure S1, and the regional LD plots for each identified locus are shown in Figure S2.

The identified variants explained only a small proportion of the phenotype variance, ranging from 0.058% for hepatitis to 0.113% for meningitis (Table S6). Out of these identified variants, 16 (55%) are rare variants with an MAF below 1%, and we show the number of individuals with certain genotypes in Table S1. Some of the associations may be misleading, as there are a few situations where the controls have both rare alleles and some cases have none (minor homozygotes, e.g., rs78111295 (FAT4), rs116306652 (CACNA1E), rs116886525 (APOA5), rs17587821 (ITPKB), rs117768315 (PCED1B) and rs570545343 (NLN)).

**Figure 1:**
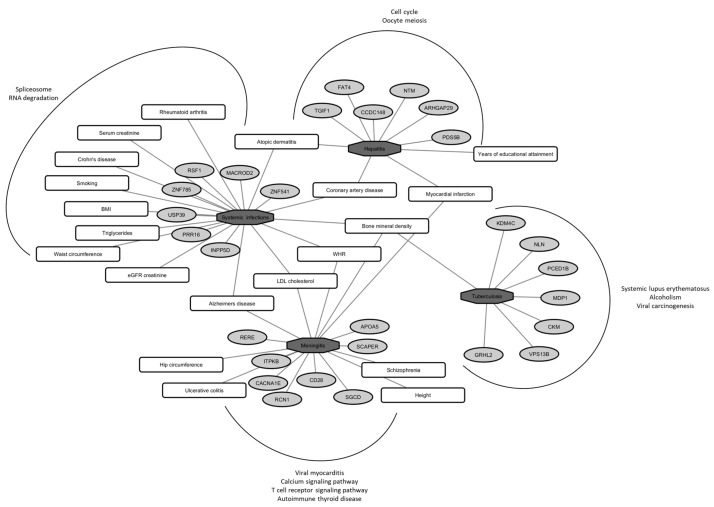
Pleiotropic network of identified loci (light grey oval shaped) with various infectious traits (dark grey octagon shaped) after a genome-wide meta-analysis of 4624 participants from the 10,001 Dalmatians biobank. Associations with various complex traits from other published GWA scans are shown in rounded rectangles, and KEGG pathway enrichment with an FDR cutoff of 1% for each of the infectious traits is depicted outside of the curved line (WHR—waist-to-hip ratio; BMI—body mass index; LDL—low-density lipoprotein; eGFR—estimated glomerular filtration rate).

Gene descriptions and their involvement in biological pathways are described in Tables S2 and S3, respectively, highlighting the transcriptome-associated pathways as the most significantly enriched pathways with systemic infections and cell cycle processes for hepatitis. In addition, these genes seem to have a role in the immune response or other cell functions related to infection susceptibility. We also checked for the association of identified loci with other traits from previously published GWA analyses, and the results are shown in Table S4, where these SNPs have been implicated in various other complex traits and diseases (e.g., atopic dermatitis, rheumatoid arthritis, Alzheimer’s disease, coronary artery disease and bone mineral density). A graphic summary of the significantly associated loci with functional enrichment and association with other complex traits is depicted in Figure 1. At the same time, detailed protein–protein interaction (PPI) partners are shown in Figure S3 for each infection-related trait for which significant GWAS loci were identified.

We investigated whether any of the previously implied variants from a meta-analysis of candidate gene studies or GWA studies on susceptibility to various bacterial and viral infectious diseases were replicated in our analyses. COVID-19 studies were excluded for this stage of the analysis, considering that for the purpose of this study, we did not have information on the COVID-19 status of the participants. The replication results are summarized in Table S5, where we show all the replicated variants with a *p*-value below 0.05.

We did not detect any significant results for the self-reported common cold or influenza frequency, probably due to the relatively small sample sizes (Table S7).

**Figure 2:**
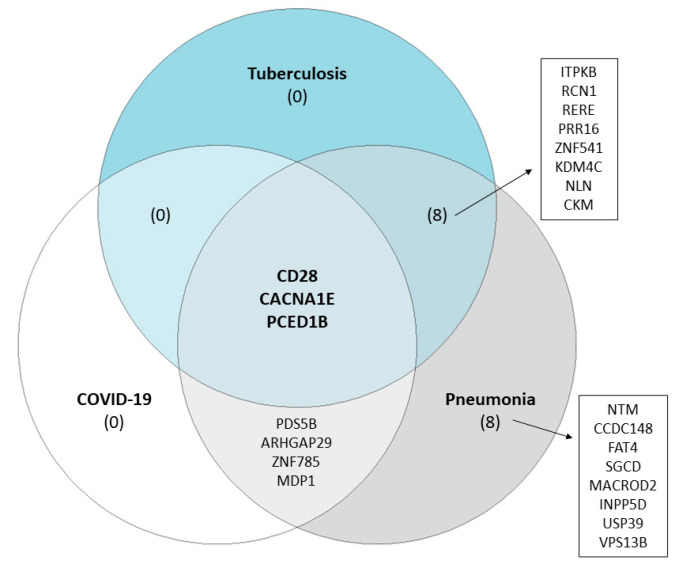
Validation of GWAS candidate loci using differentially expressed genes (FDR < 0.05) from publicly available RNA-seq studies (GEO accession IDs: pneumonia GSE196399, tuberculosis GSE94438 and COVID-19 GSE223885).

To evaluate the potential effect of these candidate rare variants, we first searched the GEO database to find publicly available RNA-seq datasets to identify differentially expressed genes (DEGs) in any of our infectious diseases for which we identified candidate SNPs—hepatitis, meningitis, tuberculosis or pneumonia. Only two RNA-seq datasets were identified as relevant based on our search criteria under the accession numbers GSE196399 (patients with severe community-acquired pneumonia vs healthy controls) and GSE94438 (tuberculosis patients vs household contacts). We supplemented this with an additional RNA-seq dataset focusing on transcriptome changes in COVID-19 patients (GSE223885). Of the 29 candidate GWAS loci, 23 were identified as DEGs in pneumonia cases, 11 in tuberculosis cases, and 7 in COVID-19 cases (Table S8). Only three genes overlap between the three studies, with CACNA1E being overexpressed, whereas CD28 and PCED1B had lower expression levels in patients with infectious disease (Figure 2). As none of the candidate GWAS variants showed eQTL in whole blood based on the GTEx data, we carried out evaluations at the gene level and identified several other SNPs in the CACNA1E, CD28 and PCED1B gene, all demonstrating lower expression in individuals with minor homozygote genotypes.

## 3. Discussion

The results of this study suggest that GWAS in biobanks may serve as a potential approach for hypothesis generation in infectious disease susceptibility. Furthermore, these results contribute to the hypothesis that the host genetic susceptibility to bacterial and viral infections in adults is polygenic and comprises common variants with low explained variance and/or “unfortunate” combinations of multiple rare variants [4,34].

Despite the small number of cases, we found several significant associations with infectious disease susceptibility, where pathway analysis revealed significant enrichment in genes and pathways involved in immune response as expected, but also in genes outside of the immune system. Most genes associated with hepatitis have been previously implicated in the pathogenesis of various liver diseases. For example, TGIF1 (TGFB-induced factor homeobox 1) is shown to be up-regulated in patients with hepatitis C virus infection [35,36], but down-regulated in fibrotic liver patients [37]. A study of patients with hepatitis B virus (HBV)-associated hepatocellular carcinoma showed that the FAT4 (FAT atypical cadherin 4) gene might act as a tumor suppressor gene, which is inactivated in cases with hepatocellular carcinoma, but also in various other human cancers [38,39]. Although the results should be taken with care, one study showed the association of hepatitis with gastric cancer [40], and the link between the two might involve the Rho GTPase activator ARHGAP29, which has been previously associated with gastric cancer [41], and associated with hepatitis in this study. Most interestingly, hepadnavirus integrates into the host genome within the gene NTM (neurotrimin) 1 h after infection [42].

Several genes that were significantly associated with meningitis in this study are involved in various neurological disorders, which is in line with ongoing and controversial studies showing that various bacterial and viral pathogens are risk factors for some neurodegenerative diseases, such as Alzheimer’s disease, Parkinson’s disease, multiple sclerosis, amyotrophic lateral sclerosis, and autism spectrum disorders [43,44,45]. Here, we report APOA5 to be associated with meningitis, while previous studies implicated various other apolipoproteins, such as APOA1 and APOE, are involved in meningitis [46,47], as well as various bacterial or respiratory infections [47,48,49], both in human and mouse studies. Additionally, the APOA1–APOC3–APOA4 gene cluster has been associated with the risk of Alzheimer’s disease [50]. CACNA1E, as identified in this study, and other voltage-gated calcium channel subunit genes have been previously associated with various neuropsychiatric disorders, such as schizophrenia, autism, and bipolar disorder [51], but also linked to the development of cortical lesions in patients with multiple sclerosis [52]. Previously performed GWA studies on other common traits also showed a significant association of the loci identified in this study with Alzheimer’s disease and schizophrenia (Table S4). We also show the association of meningitis with two genes with well-known functions in the immune response. CD28, a component of the immune system that is involved in T-cell activation, induction of cell proliferation and cytokine production and promotion of T-cell survival, was used in several animal and in vitro studies to show that CD28-deficient mice develop experimental autoimmune meningitis [53] or that the blockade of CD28 improves experimental autoimmune encephalomyelitis [54]. ITPKB (inositol-trisphosphate 3-kinase B), a well-known gene associated with the immune response, showed in animal studies its significant role in the thymocyte differentiation function of peripheral T-cells [55,56]. Functional enrichment analysis of the loci in this study associated with meningitis also showed significant enrichment in the immune response network and pathways associated with T-cell signaling (Table S3).

A single gene associated with pneumonia was APOBEC1, a member of the cytidine deaminases family, which was previously reported to play a central role in innate and adaptive immunity interplay [57], or in the delayed development of pneumocystis pneumonia in AIDS patients [58] and clearance of pneumococcal pneumonia [59].

We also identified several genes that might contribute to susceptibility to systemic infections in this study, most of which seem to have a distinct role in the immune response. RSF1 (human remodeling and spacing factor (1)) was shown to interact with SP100, which is known to have an important role in regulating the immune response to intracellular pathogens [60]. In addition, one study showed that the HBV viral protein pX interacts with RSF1 and as a result, HBV transcription is increased [61], suggesting that this gene might have a role in the life cycle of viruses whose expression is regulated at the transcription level. The role of MACROD2 (MACRO domain containing (2)) could be explained through its interaction with ARTD10, which is inducible by inflammatory and immunogenic stimuli and enhances the NF-κβ signaling pathway to increase inflammation, the innate immune response, cell survival and proliferation [62]. INPPD5, also known as SHIP1, was previously analyzed in numerous studies, and is also implicated to have a role in immune response [63]. The role of this gene is closely linked to IL10 and several important pathways [64], resulting in modifying effects in the case of the clearance of *S. aureus* and *S. pneumoniae* [65], as well as in the pathogenesis of *Pseudomonas aeruginosa* [66], Epstein–Barr virus [67], cytomegalovirus [68,69,70], or even immunity to helminth infection in mice [71].

We found a significant association between tuberculosis and KDM4C, a lysine demethylase that was also shown to be significantly associated with non-small cell lung carcinoma [72]. Another gene associated with the risk of tuberculosis was MDP1, magnesium-dependent phosphatase 1, which has also been proposed to have a role in gastric cancer [73]. This could be part of the larger pathway that explains the relationship between tuberculosis and carcinogenesis involving the CCL7-CCL2-CCR2 axis, which has a role in the immune response, tumor regulation and the WT1 gene associated with the susceptibility to tuberculosis [6]. In line with this, the KEGG pathway revealed significant enrichment in the viral carcinogenesis pathway (Table S3). Lastly, we found that PCED1B is associated with tuberculosis, in line with another recent study [74].

As most of the significantly associated variants identified in this study are rare, this finding could lead to the prediction of individualized infection disease risk based on the accumulation of such rare variants. Infections are amongst the strongest known selective pressures on our genomes [75], acting as a purifying selective pressure during childhood and historically removing the most susceptible individuals from the population [76]. On the contrary, recent generations are exposed to mandatory vaccinations, improved sanitary conditions, better pathogen control and more effective clinical treatment, so the disease-associated deleterious variants are retained and accumulate in populations. Under the assumption that the (re-)emerging epidemics will utilize similar susceptibility and pathogenesis mechanisms, identifying rare variants with strong clinical effects could become an invaluable tool for predicting an individual’s risk of being infected with specific pathogens [77].

The results of this study were obtained based on very small sample sizes for each disease in a very heterogeneous population sample, thus requiring replication in independent populations. This could partially be explained by the response bias to the voluntary participation in the 10,001 Dalmatians biobank. However, the differences might also emerge because the two sub-cohorts are isolated island populations, while the third cohort is a more heterogeneous population on the mainland. Therefore, we consider this report the initial stage for a larger scale, cross-ethnical meta-analysis to measure the effects of these and other rare variants associated with infectious disease risk. Besides the small sample size representing a major limitation, this study may also suffer from the imprecision of the traits, which were registered based on clinical examinations and hospital admittance records, or via surveys and thus relying on patient memory. The use of electronic health records with diagnosed and validated pathogens is a possible step forward, especially in biobanks that have good links with the clinical records, in order to overcome the major issues of genetic studies in the field of infectious disease susceptibility regarding sample size, harmonization of study protocols, patient characterization and improvement in the use of appropriate analytic methods [6]. Due to the pathophysiological differences between bacterial and viral infections and the limitations mentioned above (small sample sizes and trait imprecision), we opted not to include multivariate GWAS models in this study. However, future studies should build upon these initial efforts, and larger biobanks should incorporate both univariate and multivariate GWAS approaches to progress our understanding of the role of host genetic factors in host–pathogen interaction. In addition, the mechanisms described here may be population-specific, thus having a limited role in the general population. These population-specific differences are already noticeable in the demographic characteristics, with significant differences among cohorts. Due to the nature of isolated island populations, there may also be a genetic difference, which might represent bias; however, we tried to overcome this by using a meta-analysis approach and a more stringent adjusted *p*-value threshold. Nevertheless, some of the obtained rare variants in this study may provide interesting targets for future functional studies if replicated in other populations with larger sample sizes and clinically verified traits.

A potential way forward in understanding the host genetics involvement in infectious diseases susceptibility and pathophysiology could be the continuous collection of samples and data in observational studies, which could build upon the existing initiatives across Europe and elsewhere. Although the COVID-19 pandemic showed us that we are still far from fully understanding complex host–pathogen interactions, elucidating the involvement of host factors in infectious disease pathogenesis should be labelled as a high research priority, aiming to contribute to a better understanding and a possibility for the quicker translation of knowledge into clinical care.

## 4. Materials and Methods

### 4.1. Study Populations

This study is based on the Croatian biobank 10,001 Dalmatians [78,79], the most comprehensive research resource for investigating genetic, environmental and social determinants of health and disease in Croatia. The study included various measurements with over 250 disease-related quantitative traits, including medical examination, anthropometry, clinically relevant analyses, cognition, smell, taste and hearing thresholds, diet, and general lifestyle. Here, we used the data from three sub-cohorts for which we had bacterial and viral infections disease data—Korčula (N = 2833), Vis (N = 1039) and Split (N = 1012). While the first two are isolated island populations, the third originates from the second-largest city in Croatia. The data were collected in several phases, starting in 2003 up to 2014. Additionally, a follow-up postal survey was sent to participants from the sub-cohorts of Korčula and Vis in 2016, with 1082 responses (28% response rate).

The project has been approved by the Ethical Boards of the Medical Schools at the University of Zagreb and the University of Split. All participants were given information on the study aims and goals and provided informed consent before entering the study. In addition, all the methods and activities related to human subjects were performed following the relevant guidelines. Because the study took place in isolated island communities, where the identification of subjects might be easier than in the general population, a stricter personal information protection plan was utilized. This meant that we devised separate collection, storage, and management protocols of all the personal records, with exclusive access to these data only granted to the PI, who enrolled all the participants and had exclusive access to these data.

### 4.2. Genotyping and SNP Imputation

DNA was extracted from venous blood leukocytes using Nucleon BACC3 kits (Tepnel, Manchester, UK). Genotyping employed Illumina HumanHap300 v1 (Vis: 317,509 single-nucleotide polymorphisms, SNPs) and Illumina HumanHap370CNV-Quad (Korčula phase 1: 346,034 SNPs, Korčula phase 2: 719,487 SNPs; Split phase 1: 320,406 SNPs, Split phase 2: 646,888 SNPs). Illumina GenomeStudio Software v3.0 (Vis, Korčula) or v3.1 (Split) was used for genotype calling. Participants with less than 95% (Vis) or 97% (Korčula, Split) genotyping rates were removed from further analysis. SNPs with a call rate below 98%, minor allele frequency (MAF) below 1% and 0.01% for Exome Chip Markers (Korčula, Split) and *p*-value for Fisher’s exact test of the Hardy–Weinberg equilibrium (HWE) below 10–6 were also removed to remove low-quality SNPs. In addition, samples with excessive autosomal heterozygosity or gender inconsistency (based on the sex chromosomes genotypes) were also removed. This left us with a final sample size of 960 in Vis, 2700 in Korčula and 966 in Split, contributing to the total sample size of 4624 participants in this study.

Genotype data were phased using SHAPEIT v2.r873 [80] and the duohmm function [81], and imputed to the reference panel HRC v1.1 [82] using the Sanger Imputation Service. Both the phasing and imputation steps were performed separately for each genotyping platform and sub-cohort. SNPs with an imputation quality score (INFO) below 0.4 and monomorphic variants were excluded, leaving 12,468,939 SNPs for Vis, 12,382,856 SNPs for Korčula and 11,400,586 SNPs for Split available for downstream analysis.

### 4.3. Trait Definition

A total of 14 traits were used in these analyses, defined through an extensive questionnaire that catalogued the participants’ history of diseases and hospitalizations and the available medical records on these events. The following three groups of variables were identified: binary variables, binary-derived variables, and ordinal variables. The binary group was based on specific diagnoses in the subjects’ medical histories. The diagnoses investigated included tuberculosis, pneumonia, hepatitis, or meningitis. The second group consisted of derived traits that were of a broader scope, including various respiratory infections (pooled pneumonia and tuberculosis), gastrointestinal infections (pooled hepatitis and other gastrointestinal diseases), systemic infections, bacterial infections (respiratory infections and bacterial meningitis), and viral infections (hepatitis and viral meningitis). We also included appendectomy and tonsillectomy in the second group, as they are associated with infection-related traits.

The final group consists of an estimate of the infectious burden and two traits added in the follow-up survey—annual common cold frequency and influenza frequency over the last ten years. The infectious burden was defined as an individual’s burden of infectious diseases up to the date of the questionnaire and was calculated for each participant (cases coded as 1, controls coded as 0) using the following equation:

Infectious burden = (tuberculosis × 1) + (pneumonia × 1) + (hepatitis × 1) + (meningitis × 1) + (respiratory infections (tuberculosis and pneumonia cases excluded) × 1) + (gastrointestinal infections (hepatitis cases excluded) × 1) + (systemic infections × 1) + (appendectomy × 0.5) + (tonsillectomy × 0.5)

The cases of appendectomy and tonsillectomy were multiplied by 0.5, as their origin can be very heterogeneous. If a subject had the same infectious disease two or more times, their infectious burden score was multiplied by 2, to give more strength to individuals with recurrent infections, which were assumed to happen in highly susceptible individuals. The total score of infectious burden divided participants into groups coded from 0 to 4. Most participants had none or one infectious disease event, while a small number had two or more different infectious diseases during their life. Cold and influenza frequency divided participants into groups coded from 0 to 3 based on the following possible answers of having a cold/influenza: several times per year/every year in the last ten years, once a year/several times in the last ten years, less than once a year/once in the last ten years, never or almost never/never.

### 4.4. Statistical Analyses

#### 4.4.1. Genome-Wide Association Analyses

Each infection-related trait was firstly adjusted for age, gender, the first three principal components, years of schooling (ranging from 0 to 26, where elementary school is defined as 0–8, high school as 9–12, and higher education and university as higher than 13 years), socioeconomic status (measured as the total sum of the items a subject had in possession, such as plumbing, heating, phone, computer, etc., ranging from 0 to 16), number of reported different infections (in order to penalize for multiple infections), and for all respiratory infection traits (pneumonia, respiratory infections, cold frequency and influenza frequency), we additionally adjusted the model to penalize for recurrent pneumonia cases. The variables years of schooling and material status were transformed into categorical variables (3 categories for years of schooling and 4 categories for material status), in line with our previous studies [83,84,85]. Missing data were imputed based on the median value depending on the cohort, gender, and age group. For the latter purpose, participants were divided into the following age groups: 18–39, 40–64, and ≥65. To account for the population structure and familial relatedness, we calculated the kinship matrix using the ibs function from the GenABEL R package [86].

We used a logistic regression model for binary and binary-derived variables and a linear regression model for ordinal variables [87]. As the analyses were performed on imputed genetic data, we used imputed allelic dosages. All the models assumed an additive allelic effect, and the environmental residuals used for the association analysis were derived from the polygenic function in the GenABEL R package [86]. All association analyses were performed using RegScan v0.2 software [88]. Summary statistics (effect sizes, standard errors, and *p*-values) of each sub-cohorts were adjusted by the genomic control method based on GRAMMAR-gamma factors [89] (genomic inflation factor lambda and unbiased estimate of the regression coefficient) from the polygenic function in the GenABEL R package in order to account for the relatedness and population stratification.

#### 4.4.2. Meta-Analyses

Before meta-analysis, GWAS summary statistics were cleaned using the standardized QC protocol for EasyQC software v9.2 [90]. SNPs with mismatching alleles across all three sub-cohorts were excluded. We performed a fixed effect inverse-variance meta-analysis using METAL software version 2011-03-25 [91]. The genome-wide significance threshold was defined as 3.57 × 10^−9^ (standardized GWA threshold 5 × 10^−8^ divided by the number of traits analyzed) after Bonferroni correction for testing several infection-related traits, as some are highly correlated (e.g., respiratory infections and pneumonia), while the suggestive threshold was set to 5 × 10^−8^.

#### 4.4.3. Proportion of Phenotypic Variance Explained by SNPs

This was calculated for each of the identified SNPs according to the formula below (β is the β coefficient, MAF is the minor allele frequency and phvar is the phenotype variance) [92].

Phenotypic variance = β^2^ × ((2 × MAF × (1 − MAF))/phvar)

Phenotype variance was calculated based on the sample residuals adjusted for covariates. To obtain the total proportion of phenotypic variance explained by all the identified SNPs for each infection-related trait, the individual variance in each SNP was summed.

#### 4.4.4. SNP Function Annotation

SNPs within a 500 kb window around the leading SNP were extracted to annotate the region. Several tools were used, including the online tool HaploReg v4.1 (http://archive.broadinstitute.org/mammals/haploreg/haploreg.php accessed on 1 September 2022) [93] and Bioconductor biomaRt R package v2.36.1 [94,95].

To check whether the leading significant SNPs were previously associated with unrelated traits, we used the online tool PhenoScanner v1.1 (http://www.phenoscanner.medschl.cam.ac.uk/ accessed on 1 September 2022) [96] to examine the published GWAS associations. We reported the identified associations using a *p*-value threshold of 0.05 and queried for proxy SNPs with an r^2^ of 1.

#### 4.4.5. Pathway Analysis

We performed pathway analysis using the online tool STRING v10.5 (https://string-db.org/ accessed on 1 March 2023) [97] for significant infection-related traits using the genes associated with top leading SNPs. To create a network, the following default and suggested STRING settings were used: minimum required interaction score of medium confidence (0.4) and the maximum number of interactions used to limit the output to the ten best-scoring hits (both for direct and indirect interactions). We used functional enrichments for Gene Ontologies (GO) and Kegg pathways, also using STRING v10.5 with option analysis, and we reported the most significant pathways with an FDR below 1% and with a minimum of 5 genes from the network in the pathway. Networks were visualized using Cytoscape v3.7.2 [98] with GeneMANIA plugin v3.5.1 [99].

#### 4.4.6. Validation with RNA-Seq Data

The Gene Expression Omnibus database (GEO; https://www.ncbi.nlm.nih.gov/geo/ accessed on 1 March 2023) was searched for relevant publicly available RNA-seq data using the following search criteria: (1) RNA-seq data performed on human whole-blood samples; (2) studies that investigated pneumonia, hepatitis, meningitis, or tuberculosis; (3) studies published in the last year; (4) minimum ten samples per group. In addition, we searched for RNA-seq studies on COVID-19 with the same restriction search criteria. Differential gene expression analyses were performed using R package DESeq2 v1.28.1 [100]. From each included RNA-seq study, raw counts were first pre-filtered to remove genes with less than 10 read counts, normalized via DESeq2 variance stabilizing transformation and differentially expressed genes (DEGs) were identified if the false discovery rate (FDR) adjusted *p*-value was less than 0.05. Finally, the GTEx database (https://gtexportal.org/home/ accessed on 5 March 2023) was used to identify the potential eQTLs in whole blood.

## 5. Conclusions

This study identified several novel rare variants associated with susceptibility to hepatitis, meningitis, tuberculosis, and systemic infections. In turn, this demonstrates that biobanks can present a valuable resource for host genetic studies in identifying susceptibility variants to infectious diseases, despite imprecise traits and small sample sizes, which were also the main limitations of this study. Thus, we propose creating a large consortium of biobanks with access to genetic data and data on infectious diseases, ideally linked with electronic health records, to develop an extensive resource for investigating host genetics as a risk factor for susceptibility to infections. Data collection and identification of possible bias and confounders in various cohorts should be standardized across the consortium to allow for proper future analysis, and more emphasis should be given on the identification of appropriate controls (e.g., susceptible but unexposed controls, controls with undiagnosed, latent or mild infection and information on vaccination status). A harmonized consortium could identify highly penetrant rare variants that have accumulated in the modern human population. In conclusion, these efforts could elucidate a fine interplay between monogenic and polygenic effects and develop a new frontier for understanding and predicting emerging infections and epidemics.

## Figures and Tables

**Table 1:** Demographic and phenotypic characteristics of study populations.

	Vis	Korčula	Split	TOTAL	*p* *
N	960	2698	966	4624	
Gender, women; n (%)	558 (58.12)	1712 (63.45)	587 (60.77)	2857 (61.79)	0.010 ^KV^
Age in years, median (IQR)	56 (24.00)	55 (23.00)	52 (21.00)	55 (22.25)	<0.001 ^KV, KS, VS^
Years of schooling; n (%)					
Elementary school [0–8]	390 (40.63)	711 (26.35)	62 (6.42)	1163 (25.15)	<0.001 ^KV, KS, VS^
High school [9–12]	413 (43.02)	1461 (54.15)	473 (48.96)	2347 (50.76)	
University [≥13]	157 (16.35)	526 (19.50)	431 (44.62)	1114 (24.09)	
Socioeconomic status; n (%)					
1st quartile [0–8]	350 (36.46)	662 (24.54)	134 (13.87)	1146 (24.78)	<0.001 ^KV, KS, VS^
2nd quartile [9–10]	254 (26.46)	769 (28.50)	200 (20.70)	1223 (26.45)	
3rd quartile [11–12]	224 (23.33)	737 (27.32)	301 (31.16)	1262 (27.29)	
4th quartile [≥13]	132 (13.75)	530 (19.64)	331 (34.27)	993 (21.48)	
Infectious disease count; n (%)					
None	576 (60.00)	1677 (62.16)	622 (64.39)	2875 (62.18)	0.114
One	293 (30.52)	752 (27.87)	277 (28.68)	1322 (28.59)	
Two	77 (8.02)	231 (8.56)	61 (6.31)	369 (7.98)	
Three	11 (1.15)	33 (1.22)	6 (0.62)	50 (1.08)	
Four	3 (0.31)	5 (0.19)	NA	8 (0.17)	
Recurrent pneumonia cases; n (%)	9 (0.94)	16 (0.59)	1 (0.10)	26 (0.56)	0.047 ^VS^
Infectious traits; n (%)					
Tuberculosis	12 (1.25)	16 (0.59)	6 (0.62)	34 (0.74)	0.110
Pneumonia	105 (10.94)	235 (8.71)	27 (2.80)	367 (7.94)	<0.001 ^KS, VS^
Hepatitis	26 (2.71)	30 (1.11)	20 (2.07)	76 (1.64)	0.002 ^KV^
Meningitis	7 (0.73)	16 (0.59)	7 (0.73)	30 (0.65)	0.854
Respiratory infections	161 (16.77)	535 (19.83)	111 (11.49)	807 (17.45)	<0.001 ^KS, VS^
Gastrointestinal infections	39 (4.06)	55 (2.04)	33 (3.42)	127 (2.75)	0.002 ^KV^
Systemic infections	26 (2.71)	36 (1.33)	25 (2.59)	87 (1.88)	0.005 ^KV, KS^
Bacterial infections	186 (19.38)	571 (21.16)	134 (13.87)	891 (19.27)	<0.001 ^KS, VS^
Viral infections	74 (7.71)	66 (2.45)	42 (4.35)	182 (3.94)	<0.001 ^KV, KS, VS^
Appendectomy	69 (7.19)	169 (6.26)	74 (7.66)	312 (6.75)	0.273
Tonsillectomy	168 (17.50)	491 (18.20)	160 (16.56)	819 (17.71)	0.517
Infectious burden					
0	576 (60.00)	1677 (62.16)	622 (64.39)	2875 (62.18)	0.001 ^KS, VS^
0.5	151 (15.73)	385 (14.27)	168 (17.39)	704 (15.22)	
1	149 (15.52)	388 (14.38)	116 (12.01)	653 (14.12)	
1.5	41 (4.27)	164 (6.08)	38 (3.93)	243 (5.25)	
2	26 (2.71)	48 (1.78)	19 (1.97)	93 (2.01)	
2.5	8 (0.83)	22 (0.81)	2 (0.21)	32 (0.69)	
3	8 (0.83)	7 (0.26)	1 (0.10)	16 (0.35)	
3.5	1 (0.11)	3 (0.11)	NA	4 (0.09)	
4	NA	4 (0.15)	NA	4 (0.09)	
Annual cold frequency, survey response	244	771	NA	1015	
Several times per year	67 (27.46)	160 (20.75)	NA	227 (22.36)	0.274
Once a year	83 (34.01)	305 (39.56)	NA	388 (38.23)	
Less than once a year	67 (27.46)	229 (29.70)	NA	296 (29.16)	
Never or almost never	27 (11.07)	77 (9.99)	NA	104 (10.25)	
Influenza frequency in the last 10 years, survey response	197	655	NA	852	
Every year	9 (4.57)	16 (2.44)	NA	25 (2.93)	0.857
Several times	48 (24.37)	178 (27.18)	NA	226 (26.53)	
Once	57 (28.93)	180 (27.48)	NA	237 (27.82)	
Never	83 (42.13)	281 (42.90)	NA	364 (42.72)	

* post-hoc pairwise comparisons (^K^—Korčula; ^V^—Vis; ^S^—Split). Abbreviations: IQR—interquartile range; NA—not applicable.

**Table 2:** Significant results after GWA meta-analyses (filtered for the top leading SNP in ±500 kb window, Bonferroni corrected associations were significant when *p* < 3.57 × 10^−9^ or suggestive when *p* < 5 × 10^−8^, SNPs were considered not to suffer from heterogeneity based on I^2^ statistics when *p* > 0.05, with same effect direction, variants annotated to protein-coding genes and variants present in meta-analysis for all three sub-cohorts (N = 4624)).

Trait	SNP	Location	Alleles *	EAF	OR (95% CI)	*p*	Gene	Variant Type
Hepatitis	rs188290902	11:131868501	G/A	0.006	1.13 (1.09–1.16)	9.05 × 10^−12^	*NTM*	Intron
	rs72936092	2:159097061	A/G	0.004	1.16 (1.11–1.20)	8.68 × 10^−11^	*CCDC148*	Intron
	rs17077736	13:33251708	A/G	0.017	1.06 (1.04–1.08)	3.19 × 10^−10^	*PDS5B*	Intron
	rs34447953	1:94730687	A/C	0.006	1.12 (1.08–1.15)	7.99 × 10^−10^	*ARHGAP29*	Intron
	rs78111295	4:126203327	T/C	0.031	1.04 (1.03–1.06)	1.21 × 10^−9^	*FAT4*	Upstream
	rs145607180	18:3452913	T/C	0.004	1.15 (1.10–1.19)	2.97 × 10^−9^	*TGIF1*	Intron
Meningitis	rs13358188	5:155325029	G/A	0.005	1.10 (1.08–1.13)	8.46 × 10^−13^	*SGCD*	Intron
	rs17587821	1:226820605	C/T	0.013	1.06 (1.04–1.08)	4.14 × 10^−12^	*ITPKB*	3′-UTR
	rs189257688	2:204544219	T/C	0.013	1.06 (1.04–1.07)	1.31 × 10^−11^	*CD28*	Upstream
	rs188530871	15:76781806	T/C	0.006	1.08 (1.06–1.10)	7.57 × 10^−11^	*SCAPER*	Intron
	rs61878814	11:32119462	C/T	0.026	1.05 (1.03–1.06)	1.41 × 10^−10^	*RCN1*	Intron
	rs116886525	11:116671391	T/C	0.006	1.09 (1.06–1.12)	2.29 × 10^−10^	*APOA5*	Upstream
	rs35608792	1:8450425	G/A	0.023	1.04 (1.03–1.05)	4.87 × 10^−10^	*RERE*	Intron
	rs116306652	1:181673900	T/G	0.014	1.05 (1.04–1.07)	1.46 × 10^−9^	*CACNA1E*	Intron
Pneumonia	rs187624194	12:7775204	C/T	0.009	1.15 (1.10–1.20)	2.15 × 10^−8^	*APOBEC1*	Downstream
Systemic infections	rs146072725	11:77519509	T/C	0.008	1.13 (1.09–1.16)	7.50 × 10^−13^	*RSF1*	Intron
	rs142441889	20:16052044	T/G	0.006	1.13 (1.09–1.17)	1.63 × 10^−10^	*MACROD2*	Downstream
	rs76931343	5:119876729	G/A	0.005	1.14 (1.10–1.18)	3.70 × 10^−10^	*PRR16*	Intron
	rs58219087	19:48061409	C/T	0.004	1.20 (1.14–1.26)	7.57 × 10^−10^	*ZNF541*	Upstream
	rs138336976	2:234090697	A/G	0.010	1.10 (1.07–1.12)	1.26 × 10^−9^	*INPP5D*	Intron
	rs192437130	2:85840200	T/C	0.004	1.21 (1.15–1.27)	1.34 × 10^−9^	*USP39*	Intron
	rs6565193	16:30600260	C/T	0.028	1.05 (1.04–1.07)	2.38 × 10^−9^	*ZNF785*	Upstream
Tuberculosis	rs554596237	9:6953063	G/T	0.006	1.09 (1.07–1.12)	2.06 × 10^−11^	*KDM4C*	Intron
	rs145254894	14:24683304	A/C	0.011	1.07 (1.05–1.09)	1.17 × 10^−10^	*MDP1*	Missense
	rs570545343	5:65171990	A/G	0.015	1.06 (1.04–1.07)	1.24 × 10^−10^	*NLN*	Downstream
	rs117768315	12:47562000	A/G	0.010	1.06 (1.04–1.07)	1.48 × 10^−10^	*PCED1B*	Intron
	rs182320411	19:45821257	A/G	0.011	1.08 (1.05–1.10)	9.69 × 10^−10^	*CKM*	Intron
	rs140511699	8:102628803	C/A	0.008	1.07 (1.05–1.10)	2.37 × 10^−9^	*GRHL2*	Intron
	rs140782448	8:100743166	C/T	0.004	1.10 (1.07–1.13)	2.99 × 10^−9^	*VPS13B*	Intron

* Alleles: risk allele (effect allele)/other allele (non-effect allele). Abbreviations: EAF—effect allele frequency.

## Data Availability

The data analyzed during the current study are available upon reasonable requests to the corresponding author.

## Supplementary Materials

The following supporting information can be downloaded at: https://www.mdpi.com/article/10.3390/ijms24087006/s1.
