# Supplementary material for: Genome-Wide Meta-Analysis Identifies Multiple Novel Rare Variants to Predict Common Human Infectious Diseases Risk
Source: Int J Mol Sci. 2023 Apr 10;24(8):7006. doi: 10.3390/ijms24087006 (PMC10138356; doi:10.3390/ijms24087006)
Supplement: Supplementary file 1 [file ijms-24-07006-s001.zip › ijms-2323082-supplementary.pdf]

Supplementary materials

Genome-wide meta-analysis identifies multiple novel rare variants to predict common human infectious diseases risk

Figure S1. Manhattan and QQ plots of five traits with significant results after GWA meta-analyses

Figure S2. Regional LD plots of significant infection-related loci after GWA meta-analyses

Figure S3. Protein-protein interaction (PPI) networks of identified significant GWAS loci

Table S1. Number of individuals with certain genotype based on their status as controls or cases for 29 significant infection-related loci after GWA meta-analyses

Table S2. Gene annotation of significant infection-related loci after GWA meta-analyses

Table S3. Functional enrichment of significant infection-related loci after GWA meta-analyses

Table S4. Database association of previously published GWAS results with significant infection-related loci identified in this study

Table S5. Summary of replication results with previously published meta-analysis of candidate gene studies and GWAS hits

Table S6. Proportion of the phenotypic variance explained by single SNP or joined effect of all identified GWAS hits

Table S7. Results of GWAS analysis for the self-reported frequencies of common cold and influenza (survey-based responses)

Table S8. Validation of GWAS candidate loci from publicly available RNA-seq studies

a) Hepatitis

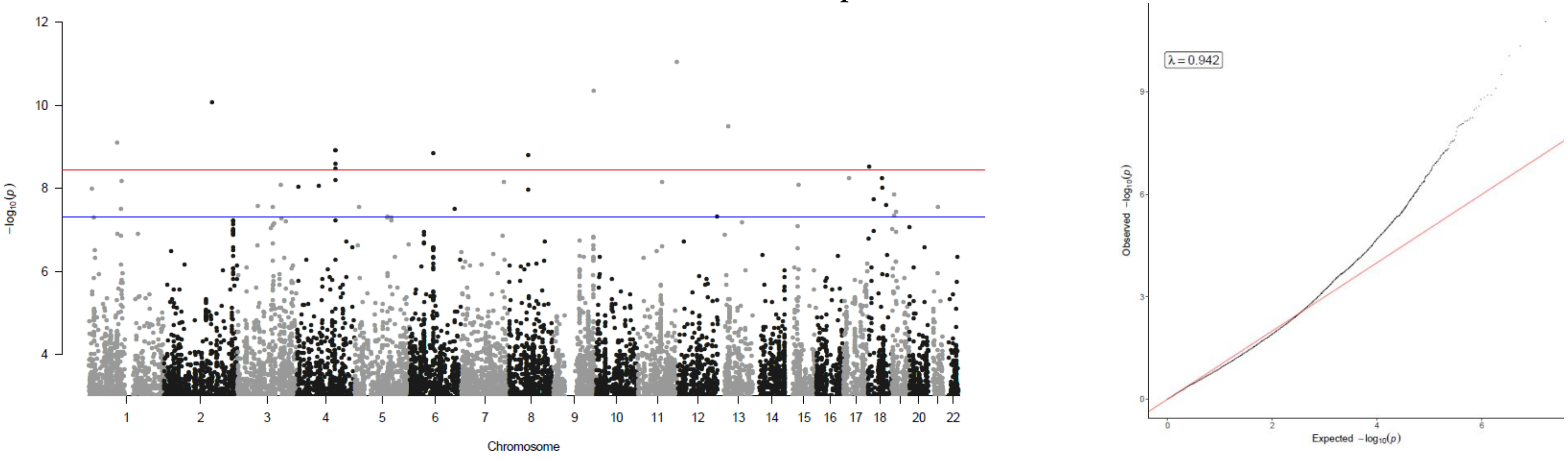

b) Meningitis

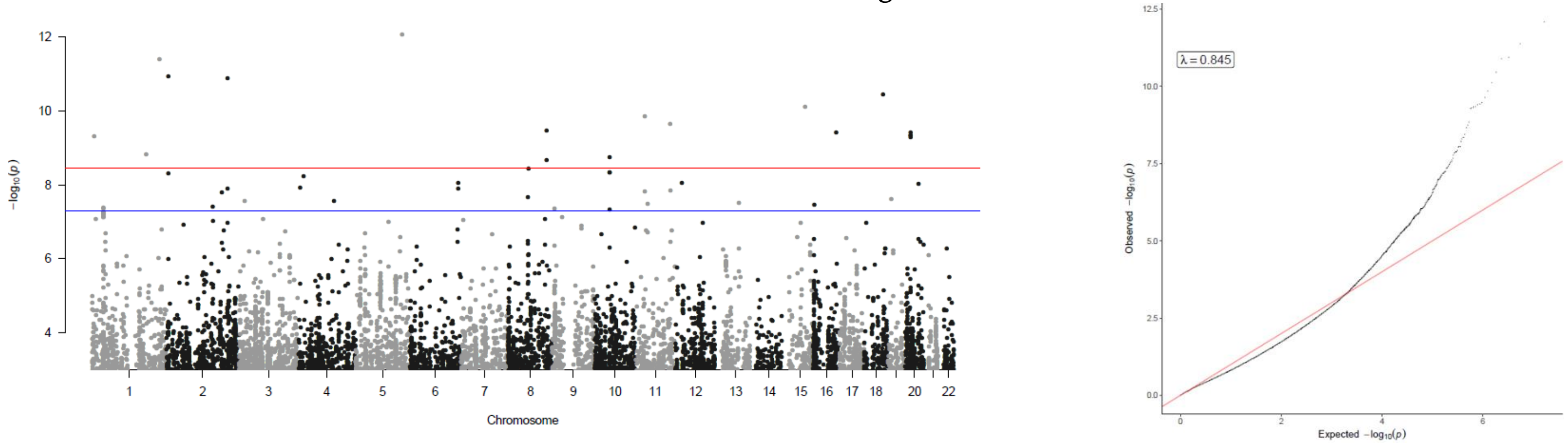

c) Pneumonia

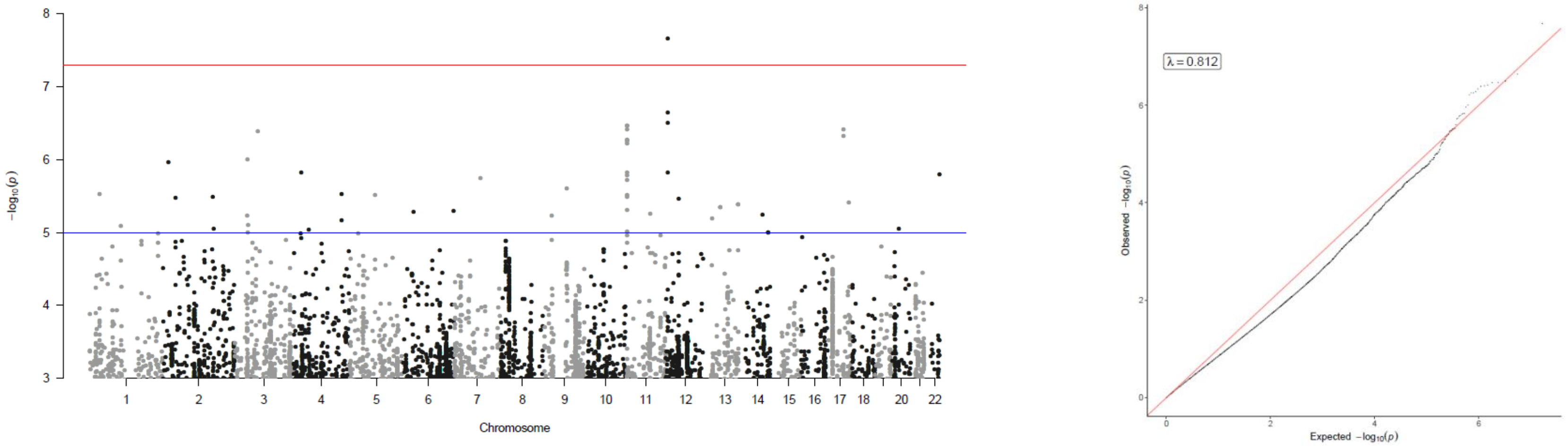

d) Systemic infections

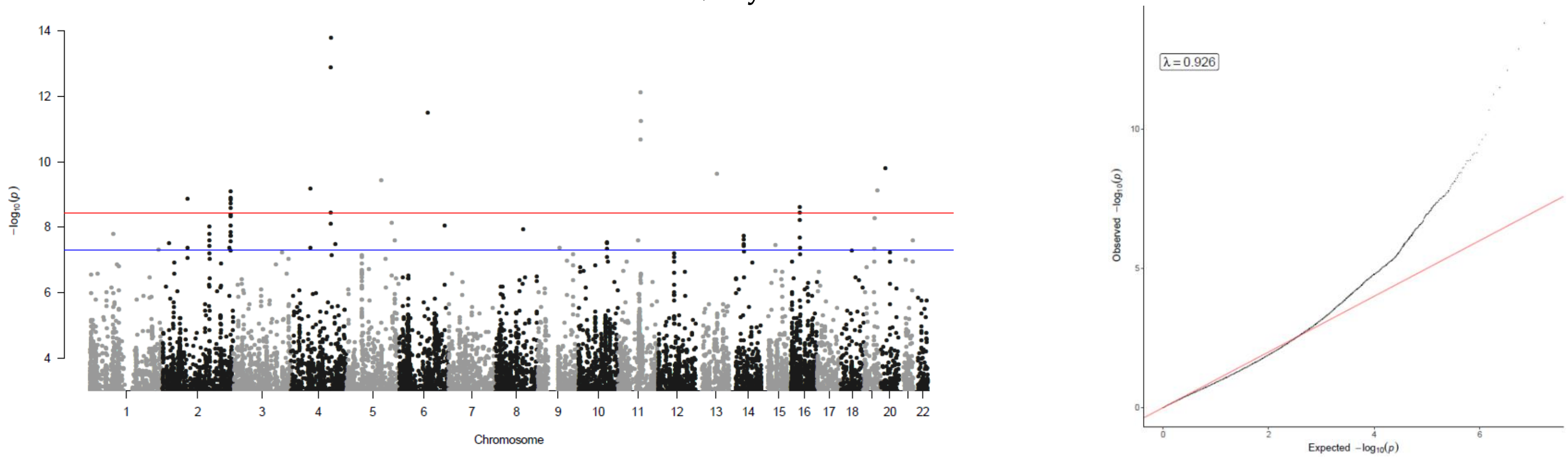

e) Tuberculosis

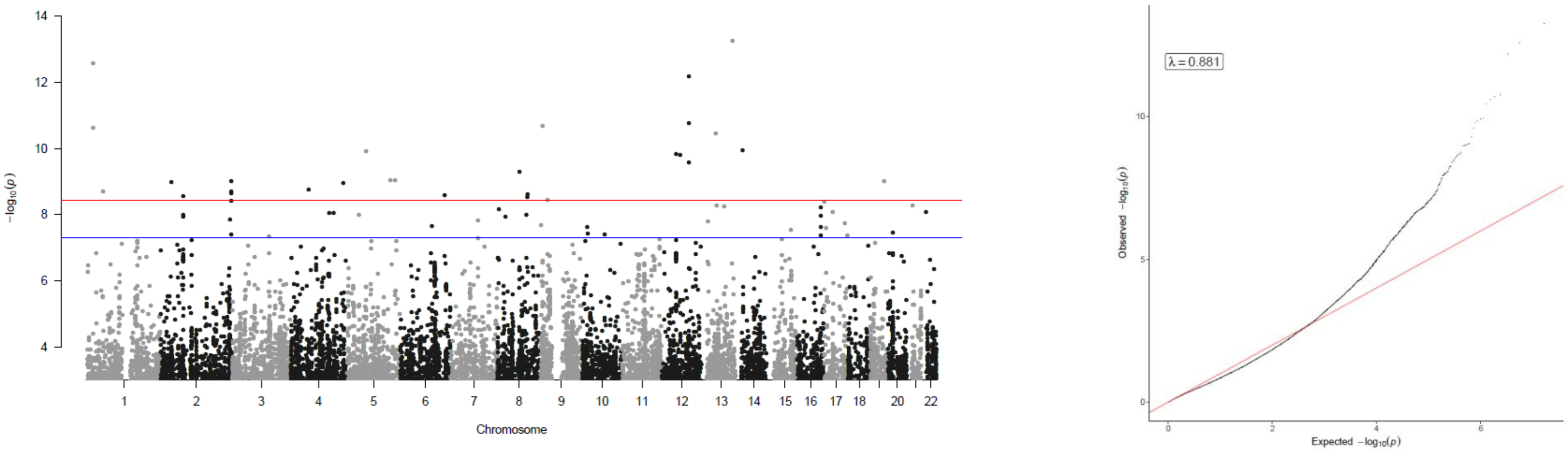

Figure S1. Manhattan and QQ plots of five traits with significant results after GWA meta-analyses with variants present in meta-analysis for all three sub-cohorts [N=4,624] (in Manhattan plots red line represent Bonferroni corrected genome-wide significant threshold 3.57×10^{-9}, and blue line represent suggestive threshold 5×10^{-08}; in QQ plots λ represent genomic inflation factor)

Hepatitis: rs188290902

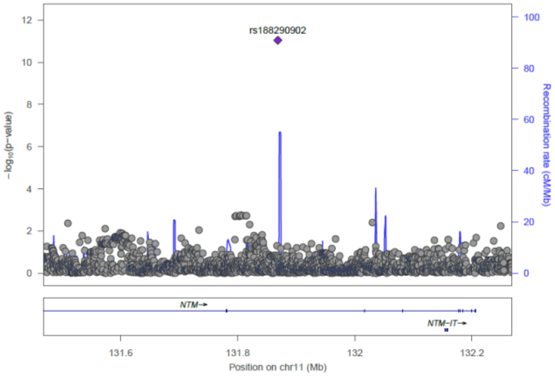

Hepatitis: rs72936092

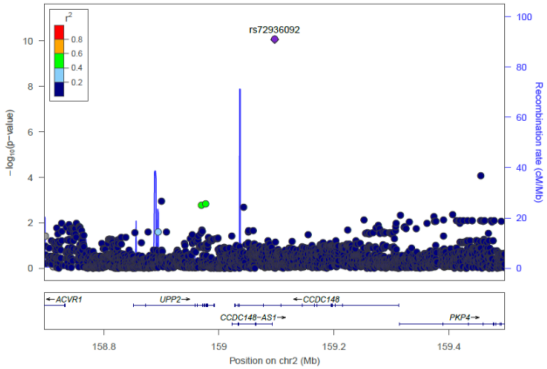

Hepatitis: rs17077736

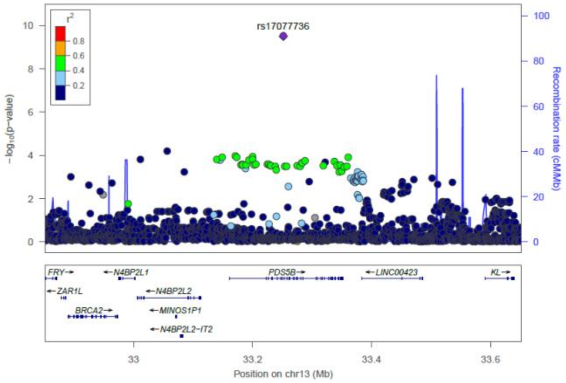

Hepatitis: rs34447953

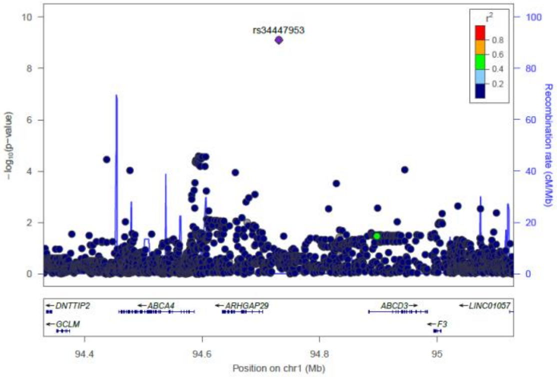

Hepatitis: rs78111295

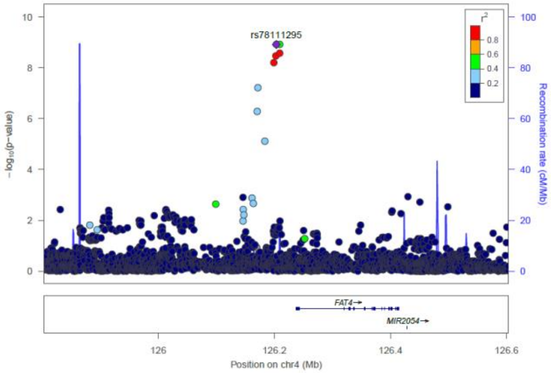

Hepatitis: rs145607180

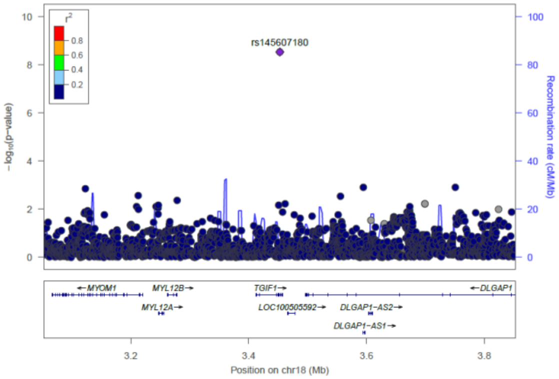

Meningitis: rs13358188

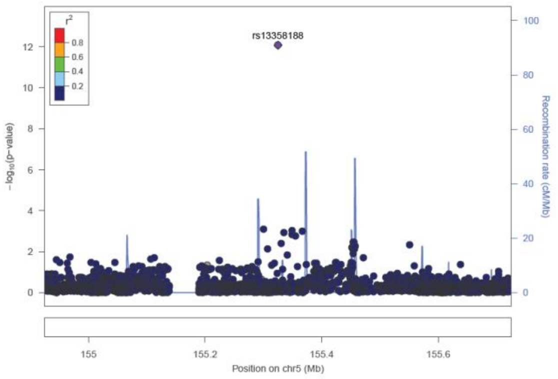

Meningitis: rs17587821

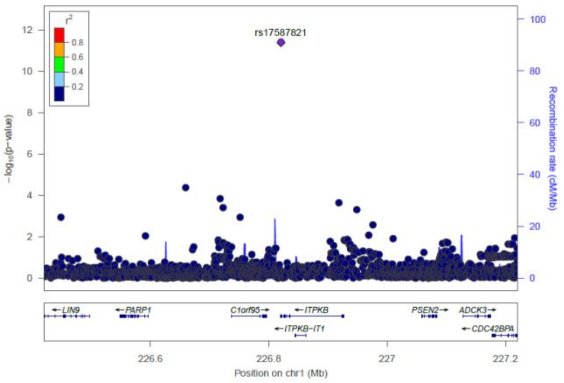

Meningitis: rs189257688

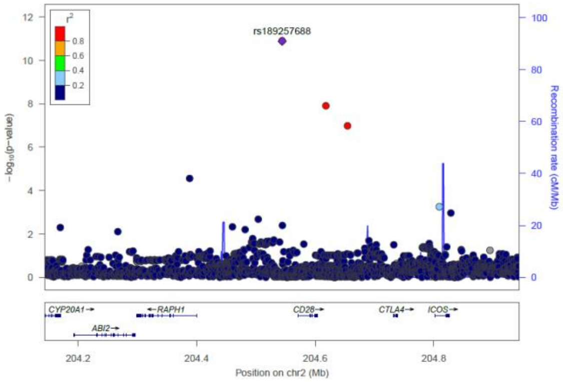

Meningitis: rs188530871

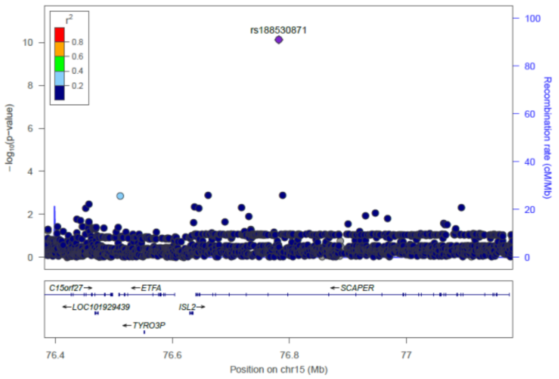

Meningitis: rs61878814

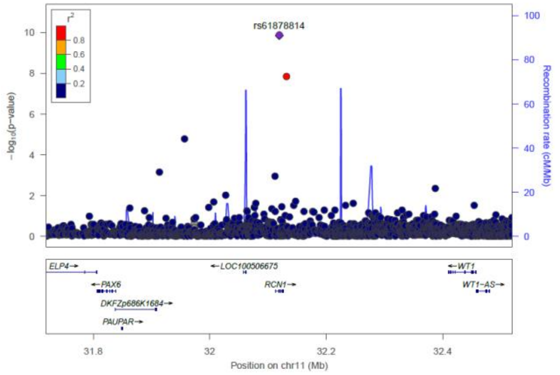

Meningitis: rs116886525

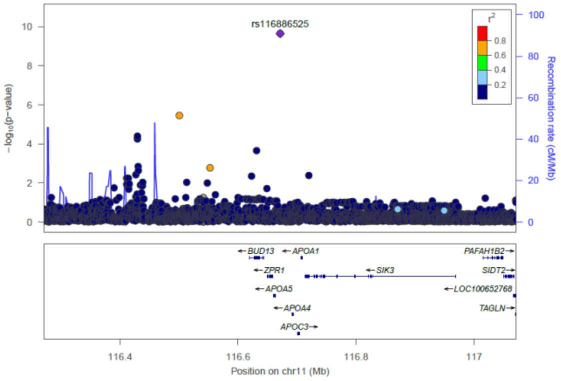

Meningitis: rs35608792

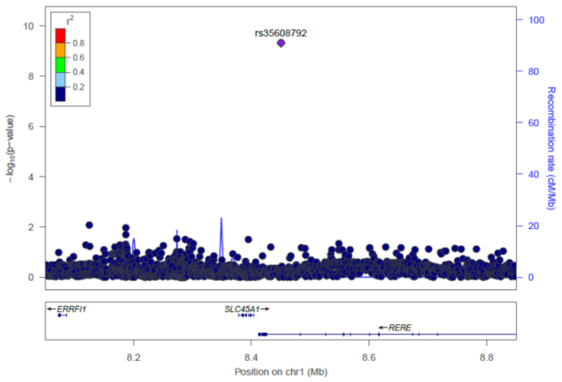

Meningitis: rs116306652

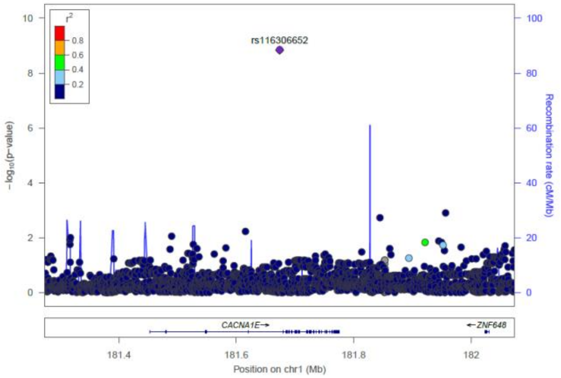

Pneumonia: rs187624194

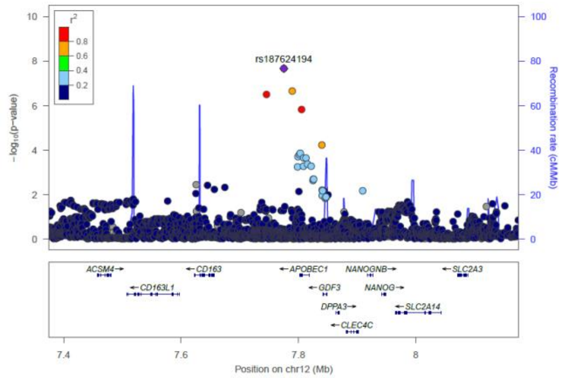

Systemic infections: rs146072725

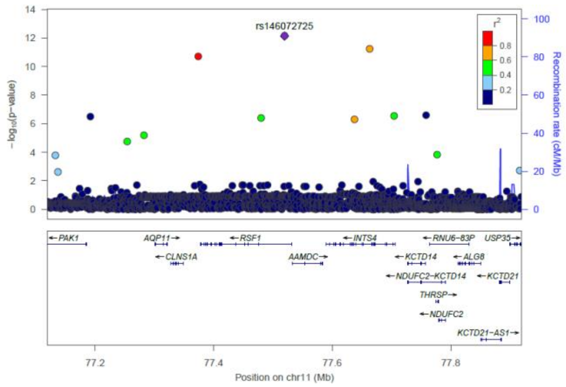

Systemic infections: rs142441889

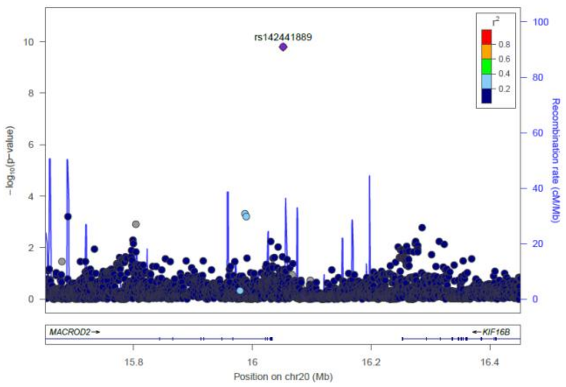

Systemic infections: rs76931343

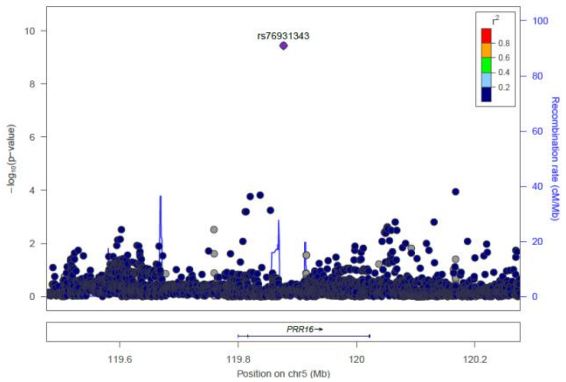

Systemic infections: rs58219087

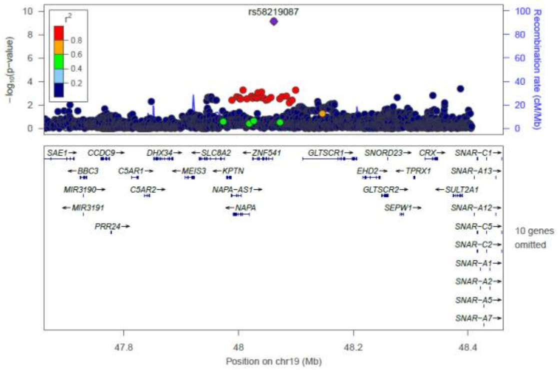

Systemic infections: rs138336976

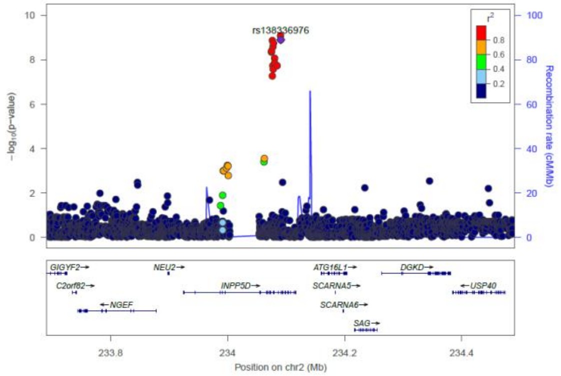

Systemic infections: rs192437130

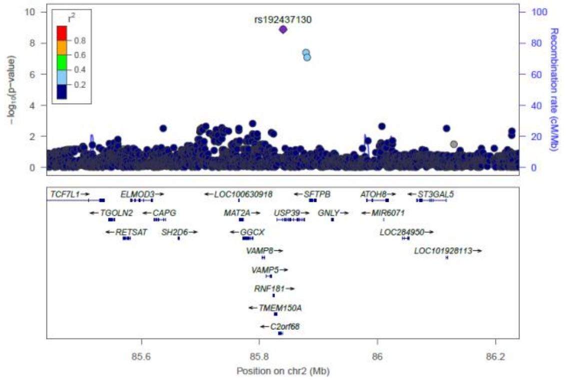

Systemic infections: rs6565193

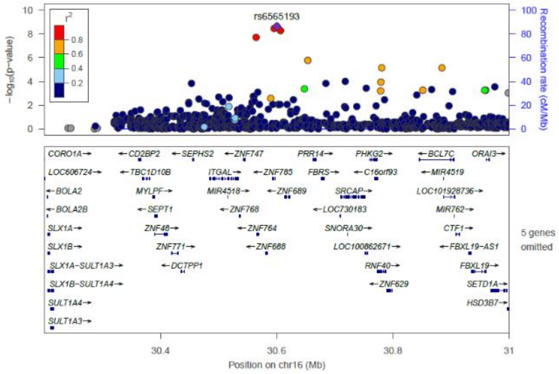

Tuberculosis: rs145254894

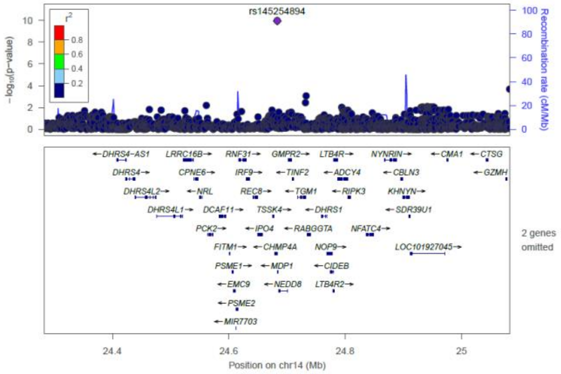

Tuberculosis: rs117768315

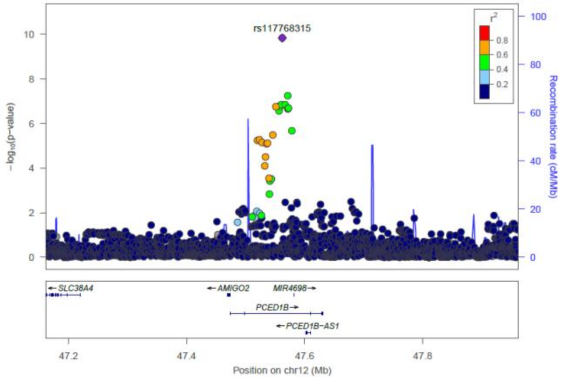

Tuberculosis: rs182320411

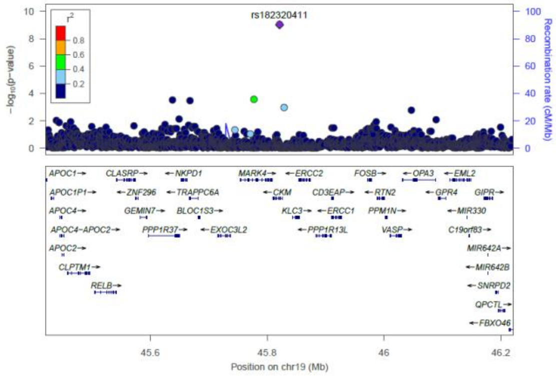

Tuberculosis: rs140511699

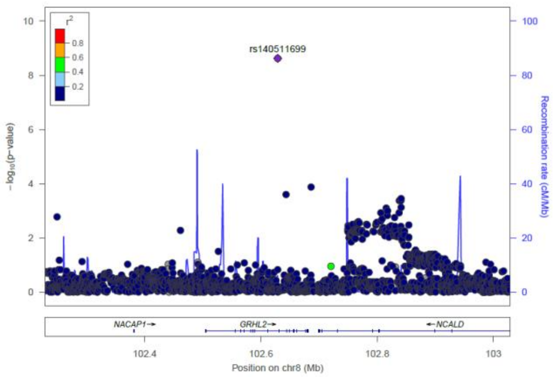

Tuberculosis: rs140782448

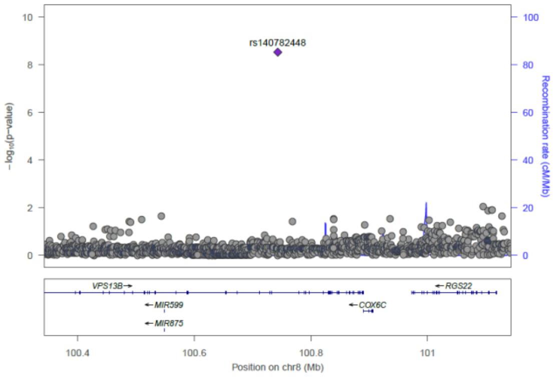

Figure S2. Regional LD plots of significant infection-related loci after GWA meta-analyses

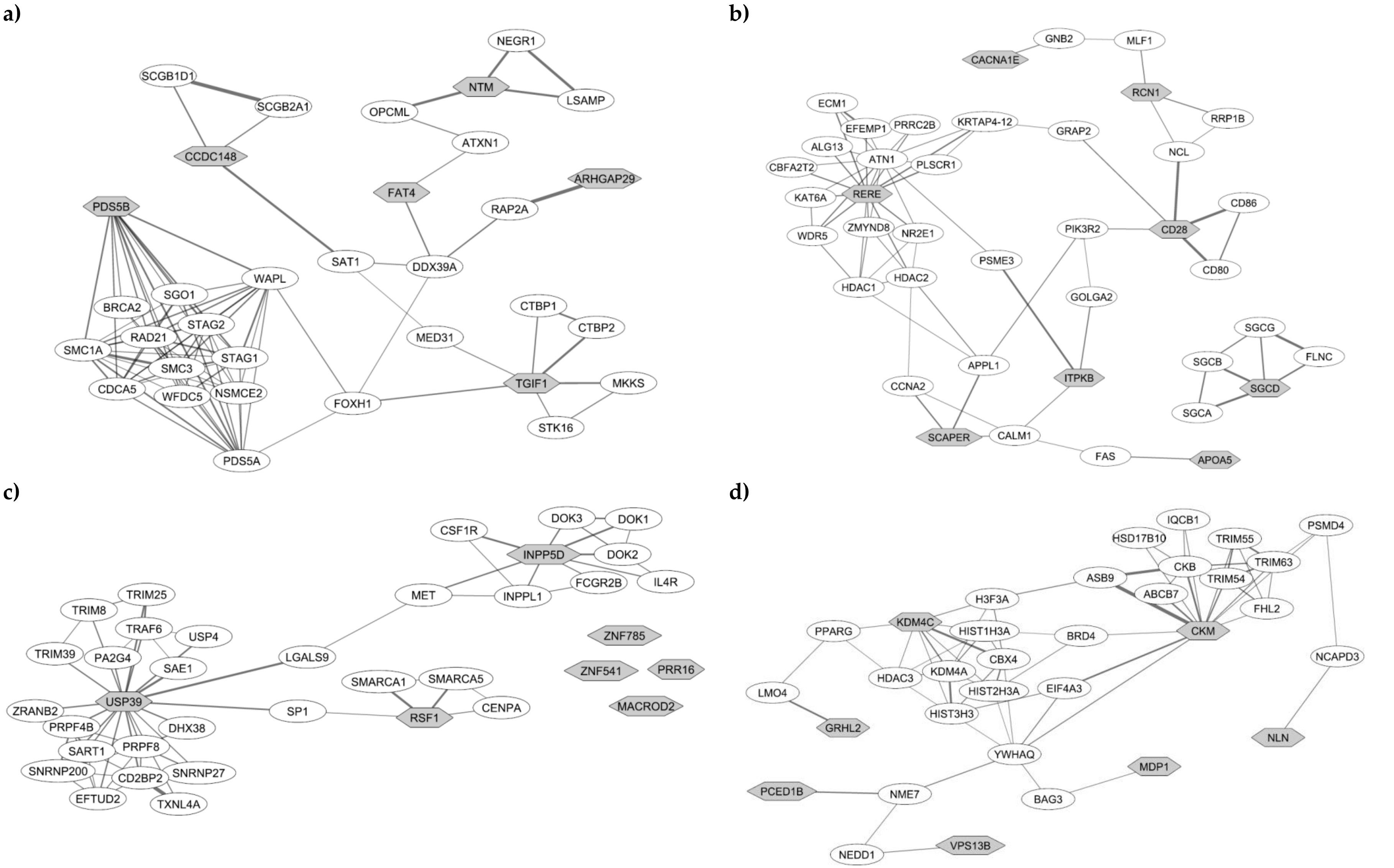

Figure S3. Protein-protein interaction (PPI) networks of identified significant GWAS loci (*grey hexagon shaped*) for a) hepatitis, b) meningitis, c) systemic infections, and d) tuberculosis (PPI partners identified as physical interactions with GeneMANIA Cytoscape plugin; top 50 proteins shown with removed nodes with less than two edges)

Table S1. Number of individuals with certain genotype based on their status as controls or cases for 29 significant infection-related loci after GWA meta-analyses (N=4,624)

Trait	SNP	Alleles[a]	Genotype[b]					
			0		1		2	
			Controls	Cases	Controls	Cases	Controls	Cases
Hepatitis	rs145607180	C/T	4524	72	26	4	0	0
	rs17077736	G/A	4398	64	150	11	2	1
	rs188290902	A/G	4496	69	54	7	0	0
	rs34447953	C/A	4496	71	54	4	0	1
	rs72936092	G/A	4515	71	35	5	0	0
	rs78111295	C/T	4280	60	267	16	3	0
Meningitis	rs116306652	G/T	4490	24	104	6	2	0
	rs116886525	C/T	4556	26	39	4	1	0
	rs13358188	A/G	4563	26	33	4	0	0
	rs17587821	T/C	4496	24	99	6	1	0
	rs188530871	C/T	4542	26	54	4	0	0
	rs189257688	C/T	4477	24	119	5	0	1
	rs35608792	A/G	4418	22	178	8	0	0
	rs61878814	T/C	4448	24	147	4	1	2
Pneumonia	rs187624194	T/C	4198	348	61	19	0	0
Systemic infections	rs138336976	G/A	4445	78	94	9	0	0
	rs142441889	G/T	4489	81	50	6	0	0
	rs146072725	C/T	4480	79	59	7	0	1
	rs192437130	C/T	4516	82	23	5	0	0
	rs58219087	T/C	4509	83	30	4	0	0
	rs6565193	T/C	4312	69	227	17	0	1
	rs76931343	A/G	4504	81	35	6	0	0
Tuberculosis	rs117768315	G/A	4493	28	97	6	2	0
	rs140511699	A/C	4541	29	51	5	0	0
	rs140782448	T/C	4560	31	32	3	0	0
	rs145254894	C/A	4510	28	82	5	0	1
	rs182320411	G/A	4534	29	58	5	0	0
	rs554596237	T/G	4557	30	35	4	0	0
	rs570545343	G/A	4447	28	143	6	2	0

[a] *Alleles: non-effect allele (major) / effect allele (minor)*

[b] *Genotype: 0 – major homozygote, 1 – heterozygote, 2 – minor homozygote*

Table S2. Gene annotation of significant infection-related loci after GWA meta-analyses

Trait	Gene	Description
Hepatitis	CCDC148	Coiled-coil domain containing 148
	TGIF1	TGFB-induced factor homeobox 1; Binds to a retinoid X receptor (RXR) responsive element from the cellular retinol-binding protein II promoter (CRBPII- RXRE).
	ARHGAP29	Rho GTPase activating protein 29; GTPase activator for the Rho-type GTPases by converting them to an inactive GDP-bound state.
	PDS5B	PDS5, regulator of cohesion maintenance, homolog B (S. cerevisiae); Regulator of sister chromatid cohesion in mitosis which may stabilize cohesin complex association with chromatin.
	FAT4	FAT tumor suppressor homolog 4 (Drosophila); Plays a role in the maintenance of planar cell polarity as well as in inhibition of YAP1-mediated neuroprogenitor cell proliferation and differentiation. Previous GWAS implication in susceptibility to mononucleosis measurement.
	NTM	Neurotrimin; Member of the IgLON family of immunoglobulin domain-containing glycosylphosphatidylinositol (GPI)-anchored cell adhesion molecules which may promote neurite outgrowth and adhesion via a homophilic mechanism. Previous GWAS implication in gut microbiome measurement.
Meningitis	APOA5	Apolipoprotein A-V; The protein encoded by this gene is an apolipoprotein that plays an important role in regulating the plasma triglyceride levels.
	SCAPER	S-phase cyclin A-associated protein in the ER; CCNA2/CDK2 regulatory protein that transiently maintains CCNA2 in the cytoplasm. Previous GWAS implication in central nervous system cancer.
	CD28	CD28 molecule; Involved in T-cell activation, the induction of cell proliferation and cytokine production and promotion of T-cell survival.
	CACNA1E	Calcium channel, voltage-dependent, R type, alpha 1E subunit; Voltage-sensitive calcium channels (VSCC) mediate the entry of calcium ions into excitable cells and are also involved in a variety of calcium-dependent processes, including muscle contraction, hormone or neurotransmitter release, gene expression, cell motility, cell division and cell death.
	RCN1	Reticulocalbin 1, EF-hand calcium binding domain; May regulate calcium-dependent activities in the endoplasmic reticulum lumen or post-ER compartment.
	RERE	Arginine-glutamic acid dipeptide (RE) repeats; Plays a role as a transcriptional repressor during development. Previous GWAS implication in tonsillectomy risk measurement and lymphocyte count.
	ITPKB	Inositol-trisphosphate 3-kinase B; The activity of this encoded protein is responsible for regulating the levels of a large number of inositol polyphosphates that are important in cellular signaling. Previous GWAS implication in susceptibility to mumps measurement.

Trait	Gene	Description
	SGCD	Sarcoglycan, delta; Component of the sarcoglycan complex, a subcomplex of the dystrophin-glycoprotein complex which forms a link between the F-actin cytoskeleton and the extracellular matrix.
Pneumonia	APOBEC1	Apolipoprotein B mRNA editing enzyme, catalytic polypeptide 1; Catalytic component of the apolipoprotein B mRNA editing enzyme complex which is responsible for the postranscriptional editing of a CAA codon for Gln to a UAA codon for stop in the APOB mRNA.
Systemic infections	USP39	Ubiquitin specific peptidase 39; Plays a role in pre-mRNA splicing as a component of the U4/U6-U5 tri-snRNP, one of the building blocks of the spliceosome.
	MACROD2	MACRO domain containing 2; The protein encoded by this gene is a deacetylase involved in removing ADP-ribose from mono-ADP-ribosylated proteins. Previous GWAS implication in Epstein-Barr virus infection, interleukin 10 and 12 measurements and TNF-related apoptosis-inducing ligand measurement.
	RSF1	Remodeling and spacing factor 1; This gene encodes a nuclear protein that interacts with hepatitis B virus X protein (HBX) and facilitates transcription of hepatitis B virus genes by the HBX transcription activator, suggesting a role for this interaction in the virus life cycle.
	INPP5D	Inositol polyphosphate-5-phosphatase; The protein functions as a negative regulator of myeloid cell proliferation and survival. Previous GWAS implication in leukocytes count.
	ZNF785	Zinc finger protein 785; May be involved in transcriptional regulation. Previous GWAS implication in basophil and eosinophil count.
	PRR16	Proline rich 16; Regulator of cell size that promotes cell size increase independently of mTOR and Hippo signaling pathways. Previous GWAS implication in response to vaccine, cytokine measurement and interleukin 2 receptor antagonist measurement.
	ZNF541	Zinc finger protein 541; Component of some chromatin remodeling multiprotein complex that plays a role during spermatogenesis.
Tuberculosis	PCED1B	PC-esterase domain containing 1B; This gene encodes a protein that belongs to the GDSL/SGNH-like acyl-esterase family whic are hydrolases thought to function in modification of biopolymers on the cell surface.
	MDP1	Magnesium-dependent phosphatase 1; Magnesium-dependent phosphatase which may act as a tyrosine phosphatase.
	CKM	Creatine kinase, muscle; The protein encoded by this gene is a cytoplasmic enzyme involved in energy homeostasis and is an important serum marker for myocardial infarction.
	NLN	Neurolysin (metallopeptidase M3 family); Hydrolyzes oligopeptides such as neurotensin, bradykinin and dynorphin A and is likely involved in the termination of the neurotensinergic signal in the central nervous system and in the gastrointestinal tract.

Trait	Gene	Description
	GRHL2	Grainyhead-like 2 (*Drosophila*); Transcription factor playing an important role in primary neurulation and in epithelial development.
	KDM4C	Lysine (K)-specific demethylase 4C; The encoded protein is a trimethylation-specific demethylase, and converts specific trimethylated histone residues to the dimethylated form which regulates gene expression and chromosome segregation. Previous GWAS implication in HIV-1 infection and susceptibility to viral and mycobacterial infections.
	VPS13B	Vacuolar protein sorting 13 homolog B (yeast); This gene encodes a potential transmembrane protein that may function in vesicle-mediated transport and sorting of proteins within the cell and may play a role in the development and the function of the eye, hematological system, and central nervous system. Previous GWAS implication in susceptibility to childhood ear infection measurement.

Table S3. Functional enrichment of significant infection-related loci after GWA meta-analyses

Trait	Database	Pathway description	Gene count	FDR*
Hepatitis	Biological Process (GO)	sister chromatid cohesion	10	2.69E-19
		chromosome segregation	14	7.11E-19
		mitotic nuclear division	14	2.01E-15
		mitotic sister chromatid segregation	10	2.29E-15
		nuclear division	14	1.87E-14
		cell division	14	5.73E-14
		mitotic cell cycle	16	7.04E-14
		nuclear chromosome segregation	10	1.56E-13
		cell cycle process	15	5.27E-11
		chromosome organization	14	1.30E-10
		cell cycle	15	1.52E-09
		single-organism organelle organization	16	2.01E-08
		cellular response to DNA damage stimulus	10	1.68E-06
		cellular component organization	17	0.00028
	KEGG	Cell cycle	8	4.86E-10
		Oocyte meiosis	7	6.67E-09
Meningitis	Biological Process (GO)	regulation of system process	12	8.64E-10
		regulation of multicellular organismal process	17	4.87E-07
		regulation of ion transport	10	3.21E-06
		system development	18	1.12E-05
		organ development	16	1.26E-05
		regulation of localization	15	1.34E-05
		regulation of transport	13	3.48E-05
		ion transport	11	4.18E-05
		multicellular organismal development	18	4.74E-05
		cell-cell signaling	10	5.55E-05
		system process	12	0.00012
		regulation of biological quality	15	0.00016
		single-organism transport	14	0.0005
		positive regulation of multicellular organismal process	10	0.00076
		single-organism developmental process	17	0.00088
		response to endogenous stimulus	10	0.001
		cell differentiation	14	0.00108
		single-multicellular organism process	18	0.00117
		regulation of cell differentiation	10	0.00126
		response to external stimulus	11	0.00143

Trait	Database	Pathway description	Gene count	FDR*
		regulation of multicellular organismal development	10	0.00158
		cell surface receptor signaling pathway	11	0.00254
		regulation of developmental process	11	0.00275
		intracellular signal transduction	10	0.00471
		regulation of response to stimulus	13	0.00632
		immune system process	10	0.00829
		nervous system development	10	0.00829
		response to organic substance	11	0.00918
	KEGG	Viral myocarditis	7	2.22E-10
		Calcium signaling pathway	6	1.61E-05
		T cell receptor signaling pathway	5	1.96E-05
		Autoimmune thyroid disease	4	4.09E-05
		Arrhythmogenic right ventricular cardiomyopathy (ARVC)	4	0.00016
		Rheumatoid arthritis	4	0.00022
		Hypertrophic cardiomyopathy (HCM)	4	0.00022
		Dilated cardiomyopathy	4	0.00022
		Allograft rejection	3	0.00043
		Graft-versus-host disease	3	0.00046
		Type I diabetes mellitus	3	0.00057
		Intestinal immune network for IgA production	3	0.0007
		Cell adhesion molecules (CAMs)	4	0.00075
		Oxytocin signaling pathway	4	0.00107
		Long-term potentiation	3	0.00165
		Amphetamine addiction	3	0.0017
		PPAR signaling pathway	3	0.00183
		Systemic lupus erythematosus	3	0.00452
		HIF-1 signaling pathway	3	0.0056
		Oocyte meiosis	3	0.00563
		Dopaminergic synapse	3	0.00871
Systemic infections	Biological Process (GO)	RNA splicing	18	1.24E-22
		mRNA processing	18	1.49E-21
		RNA splicing, via transesterification reactions	15	6.89E-20
		mRNA splicing, via spliceosome	14	3.99E-18
		mRNA metabolic process	17	1.54E-17
		ribonucleoprotein complex assembly	10	3.36E-12
		ribonucleoprotein complex biogenesis	11	6.43E-11
		RNA metabolic process	21	1.17E-09
		cellular macromolecular complex assembly	12	1.32E-09

Trait	Database	Pathway description	Gene count	FDR*
		gene expression	21	1.15E-08
		nucleobase-containing compound metabolic process	21	9.06E-08
		nucleic acid metabolic process	20	1.42E-07
		cellular component biogenesis	13	4.15E-05
		cellular macromolecule metabolic process	20	0.00063
	KEGG	Spliceosome	19	1.07E-34
		RNA degradation	8	4.24E-12
Tuberculosis	KEGG	Systemic lupus erythematosus	13	9.37E-22
		Alcoholism	13	8.09E-20
		Viral carcinogenesis	5	0.00034

** FDR: false discovery rate; GO Biological Processes with at least 10 genes are shown*

Table S4. Database association of previously published GWAS results with significant infection-related loci identified in this study

Trait	SNP	Proxy SNP	Location	r^{2*}	Trait	PMID	N	P
Hepatitis	rs17077736	rs17077736	13:33251708	1.00	Coronary artery disease	26343387	184305	0.026
	rs17077736	rs17077736	13:33251708	1.00	Myocardial infarction	26343387	167181	0.022
	rs17077736	rs17077736	13:33251708	1.00	Years of educational attainment	27225129	328917	0.019
	rs78111295	rs78111295	4:126203327	1.00	Atopic dermatitis	26482879	103066	0.029
	rs78111295	rs77905502	4:126199248	1.00	Atopic dermatitis	26482879	103066	0.029
Meningitis	rs116886525	rs116886525	11:116671391	1.00	Alzheimers disease	24162737	54162	0.021
	rs116306652	rs116306652	1:181673900	1.00	Myocardial infarction	26343387	167181	0.035
	rs61878814	rs61878814	11:32119462	1.00	Lumber spine bone mineral density	26367794	28498	0.028
	rs61878814	rs61878814	11:32119462	1.00	Schizophrenia	25056061	82315	0.011
	rs17587821	rs17587821	1:226820605	1.00	Waist hip ratio in females	23754948	346	0.008
	rs13358188	rs13358188	5:155325029	1.00	Ulcerative colitis	23128233	20672	0.011
	rs13358188	rs13358188	5:155325029	1.00	LDL cholesterol	19060906	40463	0.011
Systemic infections	rs146072725	rs146072725	11:77519509	1.00	Crohns disease	26192919	20883	0.011
	rs146072725	rs117196822	11:77373909	1.00	Crohns disease	26192919	20883	0.013
	rs138336976	rs138336976	2:234090697	1.00	Coronary artery disease	26343387	184305	0.005
	rs138336976	rs138336976	2:234090697	1.00	Atopic dermatitis	26482879	103066	0.030
	rs138336976	rs138336976	2:234090697	1.00	Rheumatoid arthritis	24390342	22515	0.010
	rs138336976	rs138336976	2:234090697	1.00	Rheumatoid arthritis	24390342	80799	0.007
	rs138336976	rs117103380	2:234090507	1.00	Coronary artery disease	26343387	184305	0.022
	rs138336976	rs117103380	2:234090507	1.00	Atopic dermatitis	26482879	103066	0.030
	rs138336976	rs117103380	2:234090507	1.00	Rheumatoid arthritis	24390342	22515	0.013
	rs138336976	rs117103380	2:234090507	1.00	Rheumatoid arthritis	24390342	80799	0.008
	rs138336976	rs12470626	2:234084655	1.00	Coronary artery disease	26343387	184305	0.011
	rs138336976	rs12470626	2:234084655	1.00	Atopic dermatitis	26482879	103066	0.029
	rs138336976	rs12470626	2:234084655	1.00	Rheumatoid arthritis	24390342	22515	0.014
	rs138336976	rs12470626	2:234084655	1.00	Rheumatoid arthritis	24390342	80799	0.009
	rs138336976	rs148594325	2:234081237	1.00	Coronary artery disease	26343387	184305	0.012
	rs138336976	rs148594325	2:234081237	1.00	Atopic dermatitis	26482879	103066	0.030

Trait	SNP	Proxy SNP	Location	r^2*	Trait	PMID	N	P
	rs138336976	rs148594325	2:234081237	1.00	Rheumatoid arthritis	24390342	22515	0.013
	rs138336976	rs148594325	2:234081237	1.00	Rheumatoid arthritis	24390342	80799	0.009
	rs138336976	rs80106733	2:234080309	1.00	Coronary artery disease	26343387	184305	0.012
	rs138336976	rs80106733	2:234080309	1.00	Atopic dermatitis	26482879	103066	0.027
	rs138336976	rs80106733	2:234080309	1.00	Rheumatoid arthritis	24390342	22515	0.014
	rs138336976	rs80106733	2:234080309	1.00	Rheumatoid arthritis	24390342	80799	0.009
	rs138336976	rs143172481	2:234080110	1.00	Coronary artery disease	26343387	184305	0.012
	rs138336976	rs143172481	2:234080110	1.00	Atopic dermatitis	26482879	103066	0.027
	rs138336976	rs143172481	2:234080110	1.00	Rheumatoid arthritis	24390342	22515	0.014
	rs138336976	rs143172481	2:234080110	1.00	Rheumatoid arthritis	24390342	80799	0.009
	rs6565193	rs111813229	16:30595710	1.00	Atopic dermatitis	26482879	103066	0.041
	rs6565193	rs111813229	16:30595710	1.00	Alzheimers disease	24162737	54162	0.049
	rs58219087	rs10221463	19:48022561	1.00	Waist circumference in females	23754948	23568	0.046
	rs58219087	rs10221463	19:48022561	1.00	Waist hip ratio in females	23754948	23649	0.045
Tuberculosis	rs117768315	rs117768315	12:47562000	1.00	Forearm bone mineral density	26367794	8143	0.039
	rs182320411	rs182320411	19:45821257	1.00	Lumber spine bone mineral density	26367794	28498	0.028

* *r^2: measure of LD (squared correlation coefficient)*

Table S5. Summary of replication results with previously published meta-analysis of candidate gene studies and GWAS hits (data extracted from relevant publications[1-7])

PMID	SNP	Gene	Previously published results		This study	
			**Trait**	**P***	**Trait**	**P**
26524966	rs1024611	CCL2	tuberculosis	1.26E-05	bacterial infections	0.02885
					infectious load	0.00831
20694014	rs4331426	intergenic	tuberculosis	6.80E-09	pneumonia	0.02275
22306650	rs2057178	3' of WT1	tuberculosis	2.57E-11	infectious load	0.00718
					systemic infections	0.00818
					tuberculosis	0.01751
25297839	rs77349273	HCP5	shingles	1.00E-08	influenza frequency	0.02655
23326239	rs2854275	HLA-DQB1	EBNA-1 antibody (mononucleosis)	2.00E-10	appendectomy	0.02441
					bacterial infections	0.00344
					infectious load	0.00975
					respiratory infections	0.00576
24162738	rs4821116	UBE2L3	chronic hepatitis B	2.00E-12	viral infections	0.04813
25802187	rs2853953	3' of HLA-C		5.00E-20	gastrointestinal infections	0.04637
	rs12614	CFB		1.00E-34	tonsillectomy	0.01991
	rs422951	NOTCH4		5.00E-16	pneumonia	0.01652
	rs378352	HLA-DOA		1.00E-23	systemic infections	0.02670
					tonsillectomy	0.01885
	rs2853953	3' of HLA-C		5.00E-20	viral infections	0.03751
	rs11866328	GRIN2A		2.00E-08	influenza frequency	0.02062
21750111	rs7453920	HLA-DQB2		1.00E-12	pneumonia	0.02886
24940741	rs9276370	5' of HLA-DQA2		2.00E-12	pneumonia	0.02373
	rs7756516	HLA-DQB2		1.00E-12	pneumonia	0.01471
28928442	rs114947103	CDHR3	childhood ear Infections	5.40E-09	systemic infections	0.03285
	rs13281988	NIPAL2--[]--KCNS2		9.84E-09	meningitis	0.00354
					systemic infections	0.02738
	rs2808290	RAB18--[]--MKX		5.09E-16	appendectomy	0.02441
					bacterial infections	0.01580

PMID	SNP	Gene	Previously published results		This study	
			Trait	P*	Trait	P
					infectious load	0.00431
					pneumonia	0.00067
					respiratory infections	0.02007
	rs35213789	AUTS2		3.75E-09	tonsillectomy	0.04644
	rs681343	FUT2		3.51E-30	meningitis	0.04170
	rs66531907	IFNL4	hepatitis A	5.70E-08	meningitis	0.02157
					systemic infections	0.04165
					viral infections	0.01539
	rs9268652	HLA	hepatitis B	3.14E-09	appendectomy	0.02276
					systemic infections	0.00742
	rs2596465	HLA	mononucleosis	2.48E-09	appendectomy	0.04751
					influenza frequency	0.04513
	rs114193679	HLA	mumps	2.23E-17	respiratory infections	0.02585
	rs3862630	ST3GAL4		1.21E-08	viral infections	0.03752
	rs516316	FUT2		9.63E-72	meningitis	0.04148
	rs2894257	HLA	positive TB test	8.16E-36	appendectomy	0.03701
					gastrointestinal infections	0.01077
					hepatitis	0.00662
					viral infections	0.00412
	rs2523591	HLA	shingles	1.74E-27	infectious load	0.03691
	rs10849448	LTBR	tonsillectomy	2.35E-35	respiratory infections	0.04521
	rs12126292	DUSP10---[]---HHIPL2		7.21E-11	gastrointestinal infections	0.02887
					tonsillectomy	0.02067
	rs1391439	TET2		2.76E-12	appendectomy	0.00728
					cold frequency	0.01461
	rs2644312	GNA12		2.73E-11	appendectomy	0.04020
	rs3184504	SH2B3		3.07E-10	tuberculosis	0.04099
	rs41543314	HLA		5.36E-21	influenza frequency	0.00458

PMID	SNP	Gene	Previously published results		This study	
			Trait	P*	Trait	P
	rs635634	ABO-[]--SURF6		8.47E-09	pneumonia	0.04675
	rs7685785	CXCL13		4.52E-10	tonsillectomy	0.01791
					viral infections	0.01366
	rs80077929	IGFBP3---[]		1.74E-15	tonsillectomy	0.04759
	rs9542155	KLHL1		1.92E-11	cold frequency	0.04407
	rs146906133	FRMD5	UTI frequency	2.02E-08	systemic infections	0.04676
30290141	rs73185306	MLC1-MOV10L1	herpesvirus 6	7.30E−66	appendectomy	0.01040
					infectious burden	0. 01831

** except of meta-analysis of candidate gene studies (first three SNPs), selected only genome-wide significant GWAS hits ($P < 5x10^{-8}$); shown only association pairs where GWAS hit in this study had $P < 0.05$*

Table S6. Proportion of the phenotypic variance explained by single SNP or joined effect of all identified GWAS hits

Phenotype	Phenotype variance	SNP	Proportion of phenotypic variance explained by	
			Single SNP	Sum of all identified SNPs
Hepatitis	0.015	rs188290902	0.012	0.058
		rs72936092	0.012	
		rs17077736	0.008	
		rs34447953	0.010	
		rs78111295	0.006	
		rs145607180	0.010	
Meningitis	0.006	rs13358188	0.015	0.113
		rs17587821	0.015	
		rs189257688	0.015	
		rs188530871	0.012	
		rs61878814	0.020	
		rs116886525	0.015	
		rs35608792	0.012	
		rs116306652	0.011	
Pneumonia	0.052	rs187624194	0.007	0.007
Systemic infections	0.017	rs146072725	0.014	0.085
		rs142441889	0.010	
		rs76931343	0.010	
		rs58219087	0.016	
		rs138336976	0.011	
		rs192437130	0.017	
		rs6565193	0.008	
Tuberculosis	0.007	rs554596237	0.013	0.090
		rs145254894	0.014	
		rs570545343	0.014	
		rs117768315	0.010	
		rs182320411	0.018	
		rs140511699	0.010	
		rs140782448	0.010	

Table S7. Results of GWAS analysis for the self-reported frequencies of common cold and influenza (survey-based responses)

Phenotype	SNP	Location	Alleles*	EAF**	OR (95% CI)	P	N	Gene	Variant type
Common cold frequency	rs34120090	14:91663685	A/G	0.069	0.67 (0.51-0.82)	5.09E-07	1015	C14orf159	intron
	rs72811464	10:77654682	C/T	0.014	0.32 (0.00-0.77)	5.62E-07	1015	C10orf11	intron
	rs77745981	7:127063300	A/G	0.125	1.35 (1.23-1.47)	6.25E-07	1015	ZNF800	intron
	rs1351361	8:63577738	A/G	0.182	0.79 (0.69-0.89)	2.11E-06	1015	NKAIN3	intron
	rs2235247	20:7912908	G/A	0.094	0.71 (0.57-0.85)	2.39E-06	1015	HAO1	intron
	rs12215418	6:18150145	C/T	0.077	0.69 (0.54-0.85)	2.87E-06	1015	TPMT	intron
	rs141486896	22:36974893	T/G	0.034	0.59 (0.36-0.81)	3.29E-06	1015	CACNG2	intron
	rs1756957	13:28446006	C/T	0.458	1.21 (1.13-1.28)	4.36E-06	1015	PDX1-AS1	intron
	rs112219086	20:5776349	T/C	0.071	0.63 (0.44-0.83)	4.89E-06	1015	C20orf196	intron
Influenza frequency	rs114363831	2:9769591	G/C	0.042	1.83 (1.60-2.06)	2.11E-07	852	YWHAQ	intron
	rs7225162	17:43036260	C/T	0.464	1.25 (1.16-1.33)	2.58E-07	852	C1QL1	downstream
	rs112040172	8:31659501	C/T	0.021	2.17 (1.85-2.48)	1.88E-06	852	NRG1	intron
	rs10097219	8:24786803	A/G	0.164	0.76 (0.65-0.88)	2.36E-06	852	NEFM	downstream
	rs75426385	22:30928956	A/G	0.058	0.64 (0.45-0.82)	2.49E-06	852	SEC14L6	intron
	rs12504682	4:7541147	G/A	0.305	0.80 (0.71-0.89)	2.68E-06	852	SORCS2	intron
	rs2153905	1:116397961	T/C	0.470	1.21 (1.13-1.29)	2.89E-06	852	NHLH2	upstream
	rs10827935	10:20347087	G/A	0.194	1.27 (1.17-1.37)	3.39E-06	852	PLXDC2	intron

* *Alleles: effect allele (minor) / non-effect allele (major)*

** *EAF: effect allele frequency*

Table S8. Validation of GWAS candidate loci from publicly available RNA-seq studies (GEO accession IDS: pneumonia GSE196399, tuberculosis GSE94438, COVID-10 GSE223885)

Trait	Gene	COVID-19		Pneumonia		Tuberculosis	
		Fold change (log2)	P	Fold change (log2)	P	Fold change (log2)	P
Hepatitis	NTM	/	/	-1.855	1.41E-02	/	/
	CCDC148	0.838	5.59E-01	0.913	6.23E-03	/	/
	PDS5B	0.448	6.47E-03	0.291	5.75E-04	-0.002	9.83E-01
	ARHGAP29	0.682	9.75E-05	1.579	2.74E-06	0.159	3.62E-01
	FAT4	0.704	4.70E-01	-2.101	3.87E-11	-0.174	3.91E-01
	TGIF1	0.262	2.47E-01	-0.228	1.18E-01	-0.036	3.49E-01
Meningitis	SGCD	-0.202	/	-1.875	4.96E-06	0.116	5.81E-01
	ITPKB	-0.429	2.05E-01	-0.538	1.16E-07	-0.134	1.04E-04
	CD28	-1.577	3.80E-09	-1.655	8.94E-11	-0.219	8.15E-04
	SCAPER	0.050	8.21E-01	-0.068	5.88E-01	0.010	8.57E-01
	RCN1	-0.327	1.05E-01	-0.487	6.78E-03	0.162	1.04E-03
	APOA5	/	/	/	/	/	/
	RERE	0.105	6.64E-01	0.775	4.81E-05	0.192	4.96E-03
	CACNA1E	4.982	1.13E-04	4.465	1.14E-21	0.759	1.11E-07
Pneumonia	APOBEC1	/	/	/	/	/	/
Systemic infections	RSF1	-0.043	8.05E-01	0.074	4.17E-01	-0.028	6.89E-01
	MACROD2	0.167	7.92E-01	-1.157	3.79E-03	-0.231	1.52E-01
	PRR16	0.827	1.94E-01	1.564	1.45E-05	0.817	2.01E-06
	ZNF541	-1.144	1.79E-01	-2.086	6.94E-15	-0.276	1.05E-02
	INPP5D	0.414	8.90E-02	0.570	1.90E-05	0.018	7.72E-01
	USP39	0.036	8.70E-01	0.356	8.91E-12	0.002	9.48E-01
	ZNF785	-0.865	9.25E-05	-0.947	7.54E-09	-0.085	9.73E-02
Tuberculosis	KDM4C	0.200	5.42E-01	-0.472	3.92E-11	-0.088	2.22E-02
	MDP1	-0.500	1.94E-03	1.177	3.22E-04	0.128	1.12E-01
	NLN	-0.034	9.13E-01	-0.343	4.41E-02	0.204	3.29E-04

Trait	Gene	COVID-19		Pneumonia		Tuberculosis	
		Fold change (log2)	P	Fold change (log2)	P	Fold change (log2)	P
	PCED1B	-1.094	4.15E-14	-1.207	8.27E-10	-0.132	2.49E-02
	CKM	0.184	8.49E-01	1.160	1.40E-03	0.670	1.74E-04
	GRHL2	0.163	8.71E-01	/	/	/	/
	VPS13B	0.259	1.55E-01	0.487	2.89E-05	-0.013	8.45E-01

References:

1. Patarcic, I. *et al.* The role of host genetic factors in respiratory tract infectious diseases: systematic review, meta-analyses and field synopsis. *Sci Rep* **5**, 16119, doi:10.1038/srep16119 (2015).
2. Tian, C. *et al.* Genome-wide association and HLA region fine-mapping studies identify susceptibility loci for multiple common infections. *Nature communications* **8**, 599, doi:10.1038/s41467-017-00257-5 (2017).
3. Luo, Y. *et al.* Early progression to active tuberculosis is a highly heritable trait driven by 3q23 in Peruvians. *Nature communications* **10**, 3765, doi:10.1038/s41467-019-11664-1 (2019).
4. Liu, S. *et al.* Genomic Analyses from Non-invasive Prenatal Testing Reveal Genetic Associations, Patterns of Viral Infections, and Chinese Population History. *Cell* **175**, 347-359 e314, doi:10.1016/j.cell.2018.08.016 (2018).
5. Lees, J. A. *et al.* Joint sequencing of human and pathogen genomes reveals the genetics of pneumococcal meningitis. *Nature communications* **10**, 2176, doi:10.1038/s41467-019-09976-3 (2019).
6. Omae, Y. *et al.* Pathogen lineage-based genome-wide association study identified CD53 as susceptible locus in tuberculosis. *Journal of human genetics* **62**, 1015-1022, doi:10.1038/jhg.2017.82 (2017).
7. Zheng, R. *et al.* Genome-wide association study identifies two risk loci for tuberculosis in Han Chinese. *Nature communications* **9**, 4072, doi:10.1038/s41467-018-06539-w (2018).
